# Elimination of Foreign Sequences in Eukaryotic Viral Reference Genomes Improves the Accuracy of Virome Analysis

**DOI:** 10.1128/msystems.00907-22

**Published:** 2022-10-26

**Authors:** Junjie Chen, Yue Sun, Xiaomin Yan, Zilin Ren, Guoshuai Wang, Yuhang Liu, Zihan Zhao, Le Yi, Changchun Tu, Biao He

**Affiliations:** a Changchun Veterinary Research Institute, Chinese Academy of Agricultural Sciences, Changchun, Jilin Province, China; b Jiangsu Co-innovation Center for Prevention and Control of Important Animal Infectious Diseases and Zoonosis, Yangzhou University, Yangzhou, Jiangsu Province, China; Princeton University

**Keywords:** eukaryotic virome, viral disease diagnosis, host contamination, database contamination, foreign sequences

## Abstract

Widespread in public databases, foreign contaminant sequences pose a substantial obstacle in genomic analyses. Such contamination in viral genome databases is also notorious but more complicated and often causes questionable results in various applications, particularly in virome-based virus detection. Here, we conducted comprehensive screening and identification of the foreign sequences hidden in the largest eukaryotic viral genome collections of GenBank and UniProt using a scrutiny pipeline, which enables us to rigorously detect those problematic viral sequences (PVSs) with origins in hosts, vectors, and laboratory components. As a result, a total of 766 nucleotide PVSs and 276 amino acid PVSs with lengths up to 6,605 bp were determined, which were widely distributed in 39 families with many involving highly public health-concerning viruses, such as hepatitis C virus, Crimean-Congo hemorrhagic fever virus, and filovirus. The majority of these PVSs are genomic fragments of hosts including humans and bacteria. However, they cannot simply be regarded as foreign contaminants, since parts of them are results of natural occurrence or artificial engineering of viruses. Nevertheless, they severely disturb such sequence-based analyses as genome annotation, taxonomic assignment, and virome profiling. Therefore, we provide a clean version of the eukaryotic viral reference data set by the removal of these PVSs, which allows more accurate virome analysis with less time consumed than with other comprehensive databases.

**IMPORTANCE** High-throughput sequencing-based viromics highly depends on reference databases, but foreign contamination is widespread in public databases and often leads to confusing and even wrong conclusions in genomic analysis and viromic profiling. To address this issue, we systematically detected and identified the contamination in the largest viral sequence collections of GenBank and UniProt based on a stringent scrutiny pipeline. We found hundreds of PVSs that are related to hosts, vectors, and laboratory components. By the removal of them, the resulting data set greatly improves the accuracy and efficiency of eukaryotic virome profiling. These results refresh our knowledge of the type and origin of PVSs and also have warning implications for viromic analysis. Viromic practitioners should be aware of these problems caused by PVSs and need to realize that a careful review of bioinformatic results is necessary for a reliable conclusion.

## INTRODUCTION

Emerging infectious diseases (EIDs), especially viral ones, pose a serious threat to public health, severely challenging global security, social economy, and human life (1). For example, the ongoing COVID-19 and monkeypox have caused great social panic and economic losses worldwide, requiring global collaboration to contain them (2, 3). Rapid and accurate diagnosis of EID is a prerequisite for timely formulation and implementation of prevention and control measures. High-throughput sequencing (HTS)-based metagenomics is a promising approach for rapid diagnosis of EID because it does not require *a priori* information and is capable of identifying a comprehensive spectrum of potential agents, especially novel ones, by a single test (4, 5). Metagenomic diagnosis highly depends on reference database-based sequence analysis. Thus, a high-quality reference database with complete representativeness, functional robustness, and informational accuracy provides an important guarantee of diagnostic reliability.

However, of particular worry is that foreign contamination is very common in those public databases (6–9). For example, human sequences were usually found to contaminate the genome databases of bacteria, plants, and fish (6, 10). Merchant et al. found microbial sequences in cow genomes, but the verification indicated that such contamination was caused by multiple sequences of Neisseria gonorrhoeae that were actually derived from the cow or sheep genomes (8). Notably, a large-scale search has identified contamination of more than 2,000,000 exogenous sequences in the RefSeq, GenBank, and nr databases across bacteria, archaea, fungi, metazoans, plants, and other eukaryotes (7). These contaminating sequences can result in confusing and even wrong conclusions when these databases are used for alignment-based sequence analyses, such as genome annotation, evolutionary analysis, horizontal gene transfer prediction, and metagenome profiling (7, 9).

Foreign contamination is also notorious in viral reference sequence databases, but it is much more complicated than in databases of other organisms and has never been systematically examined, because some viruses can integrate their genomes into their hosts, making it very hard to distinguish contamination from genuine integration (7). Besides, there exist other factors affecting the quality of viral sequence databases, such as gene misannotation, lineage misclassification, and genome misassembly. Here, we term those reference sequences related to the above factors “problematic viral sequences” (PVSs), because they often cause problematic results in viral metagenomic analyses. For instance, our virome studies of sick and healthy animals over the past decade frequently encountered false-positive results for a wide range of such pathogenic viruses as bluetongue virus, bovine viral diarrhea virus (BVDV), and hepatitis C virus (HCV), which were finally verified to be bacterial or host genomic fragments. This phenomenon also occurred widely in other virome analyses (11–14). For example, African swine fever virus was surprisingly found in a bat virome (14), which was most likely due to the misannotation of host sequence, because African swine fever virus is particularly host specific and infects only swine (15). Additionally, viromic results were often contaminated by laboratory component-derived (LCD) sequences. The LCD sequences, such as those of parvovirus-like hybrid virus (16), xenotropic murine leukemia virus-related virus (17), and human endogenous retrovirus H (18), are technically viral but are often carried by nucleic acid extraction spin columns, biologicals, or experimental performers, thus easily contaminating samples (11, 12, 16–18). Those false-positive results severely weaken the accuracy of viral metagenomic detection, greatly confuse and mislead researchers, and usually require well-trained practitioners to discriminate them, hence posing a substantial obstacle to the popularization and application of virome-based analysis and diagnosis.

To address these issues, we conducted a systematic screening of PVSs hidden in the largest viral nucleotide (nt) and amino acid (aa) sequence collections using a consecutive scrutiny pipeline, which advances an understanding of the type and origin of these PVSs, highlighting the necessity for a quality check of sequences before submitting them to a public database. For convenience, we provide the pipeline code and a clean version of eukaryotic viral reference sequences, which is expected to favor fields like EID diagnosis, new virus identification, virome analysis, and other virologic studies.

## RESULTS

### The scrutiny pipeline overview.

Based on our experience of animal virome analyses (18–23), the review of other viromic publications (11–14, 16, 17), and the contamination screening of other databases (6–10, 24), we concluded that PVSs are related to hosts, vectors, laboratory components, and misclassification. Thus, we established a consecutive scrutiny pipeline, which is composed of five parts and managed using Snakemake (see Fig. S1 in the supplemental material). Because we aim at diagnosis of viral diseases and discovery of eukaryotic viruses, the first preliminary filtration step removes sequences of viruses infecting bacteria, archaea, fungi, or microorganisms or those shorter than 200 bp. The second step is host genome scrutiny, enabling us to detect sequences with origins of 28 mammalian, avian, and arthropod species, covering humans, domestic animals, and natural hosts and vectors, which are the predominant targets in virome analyses. The following step is to detect sequences derived from backbones or nonviral functional cassettes of vectors using two rounds of searches. Moreover, we noted that some viral reference sequences have incorrect lineage definitions, especially at high taxonomic levels, which is an important source of misannotation in virome analyses. Thus, we employed an exhaustive all-against-all comparison to identify misclassified sequences. Finally, we close the pipeline with multiple checks to detect LCD sequences using viral metagenomic annotation of 15 raw data sets that originated from different viromic studies spanning humans and other animals throughout the world. In order to avoid sacrificing the database’s representativeness, we used very strict criteria in these steps to prevent sequences from being misclassified as PVSs. For example, a blast search is considered positive in a certain step only if the alignment achieves an E value of ≤1e−50 and is longer than 500 (see Materials and Methods).

10.1128/msystems.00907-22.2 FIG S1 Overview of bioinformatic pipeline (left column) that is composed of heterogeneity scrutiny (upper) and finalization and assessment (lower); the right column indicates the sequence number in databases corresponding to each treatment. The genomic ID of hosts used in host genome scrutiny is shown in the bracket next to the species. Download FIG S1, TIF file, 1.7 MB. Copyright © 2022 Chen et al. 2022 Chen et al. https://creativecommons.org/licenses/by/4.0/ This content is distributed under the terms of the Creative Commons Attribution 4.0 International license.

### Host sequences are predominant in PVSs.

The viral division (gbvrl) of GenBank is the largest resource of eukaryotic viral nt sequences and has been widely used in virological research, even to construct other specialized subdatabases (25, 26), from which the Viral Genome Resources are derived to serve as a set of high-quality curated viral reference genomes and their validated genomic neighbors but lacking the full spectrum of viral diversity (27). As of 4 March 2021, gbvrl and the Viral Genome Resources have archived 3,316,373 and 288,226 nt sequences, respectively. They overlapped by 263,895 sequences; hence, we added the remaining 24,331 sequences of the Viral Genome Resources into gbvrl, which resulted in a preliminary data set (PDS) of 3,340,704 sequences. The PDS was subjected to PVS detection using the consecutive scrutiny pipeline. The preliminary filtration removed 91,549 sequences that contribute little to eukaryotic virome profiling of animals. After four rounds of scrutiny, we identified and deleted 766 nt PVSs from PDS (Data Set S1).

10.1128/msystems.00907-22.9 DATA SET S1 Details of the 766 nt PVSs and 267 aa PVSs identified in this study. Download Data Set S1, XLSX file, 0.2 MB. Copyright © 2022 Chen et al. 2022 Chen et al. https://creativecommons.org/licenses/by/4.0/ This content is distributed under the terms of the Creative Commons Attribution 4.0 International license.

**FIG 1:**
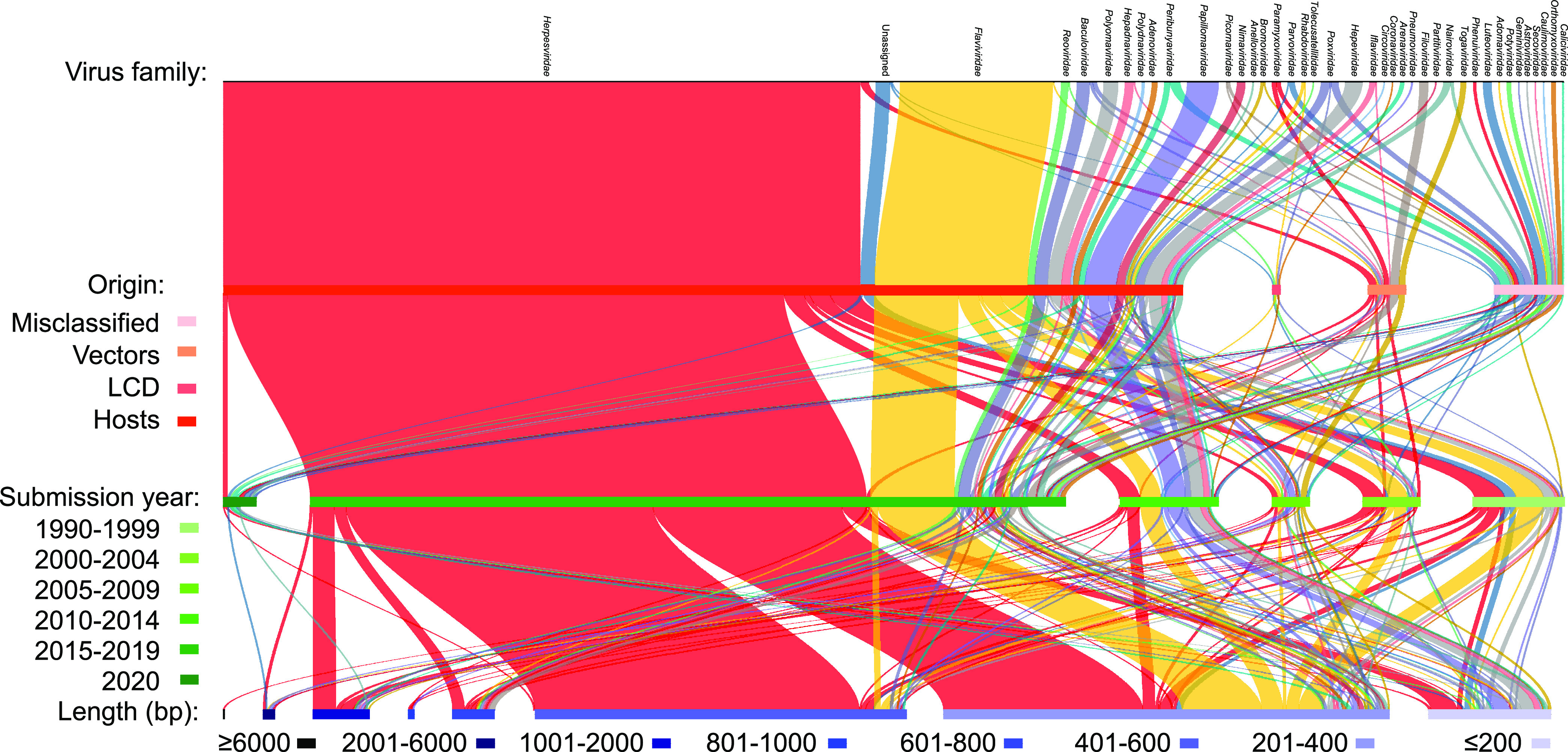
Summary of the 766 PVSs. They come from 39 viral families and are classified into misclassification, vectors, LCD, and hosts, with submission years of 1993 to 2021 and lengths of up to 6,605 bp.

These PVSs came from 39 viral families and viruses unclassified at the family level, with the majority being *Herpesviridae* (59.9%), followed by *Flaviviridae* (14.0%) (Fig. 1). They were either full-length sequences (14.5%) or chimeric fragments (85.5%) within viral genomes (Data Set S1). Host PVSs were predominant (86.9%) and were detected in 24 viral families (Fig. 1). They were related to humans and other animals, including nonhuman primates, bovines, canines, avians, rodents, bats, arthropods, etc., and even bacteria (Data Set S1). PVSs within different viral families are prone to be dominated by a certain origin, e.g., almost all PVSs within *Herpesviridae* (96.3%) and *Flaviviridae* (99.1%) were associated with host genomes, while *Togaviridae* and *Filoviridae* PVSs were all vector sequences (Fig. 1). In addition, we found that a substantial number of host PVSs (*n* > 51) submitted since 2015 were probably caused by the misassembly of Illumina reads (Text S1). The majority (80.7%) of these PVSs were ≤600 bp, with a few within the families *Papillomaviridae* (*n* = 3), *Paramyxoviridae* (*n* = 1), *Flaviviridae* (*n* = 1) and *Herpesviridae* (*n* = 3) exceeding 2,000 bp and one PVS of *Herpesviridae* even reaching 6,605 bp (Fig. 1 and Data Set S1).

10.1128/msystems.00907-22.1 TEXT S1 Details for scrutiny pipeline of viral protein sequences and for accuracy and efficiency comparisons between three reference databases in virome profiling. Download Text S1, DOCX file, 0.02 MB. Copyright © 2022 Chen et al. 2022 Chen et al. https://creativecommons.org/licenses/by/4.0/ This content is distributed under the terms of the Creative Commons Attribution 4.0 International license.

Regarding viral aa reference sequences, we retrieved all sequences under the Taxonomy of Viruses in UniProtKB (version 2021_03). UniProtKB is mainly based on the translation of genome sequences submitted to the International Nucleotide Sequences Database Collaboration (INSDC) source databases and also supplemented by genomes sequenced and/or annotated by other academic groups, making it the most comprehensive set of protein sequences (28). The aa sequence scrutiny was summarized in Data Set S1. In general, UniProt aa sequences have fewer PVSs than GenBank nt sequences, in that translation itself is a recognized validation method of viral genomes. Furthermore, foreign insertion often occurs as a flanking sequence in the untranslated region at the terminus of nt sequence. Finally, a total of 267 PVSs were detected, with most being counterparts in nt scrutiny (Data Set S1); hence, they will not be discussed below.

### The causation of PVS occurrence: natural versus artificial.

Contamination is very common in nonviral public databases and is caused by the foreign DNA of other species and even species from other kingdoms (6–10). However, we found that these PVSs cannot simply be considered foreign contaminants but rather should be ascribed to natural (na), intentionally artificial (ia), and unintentionally artificial (ua) occurrences.

**FIG 2:**
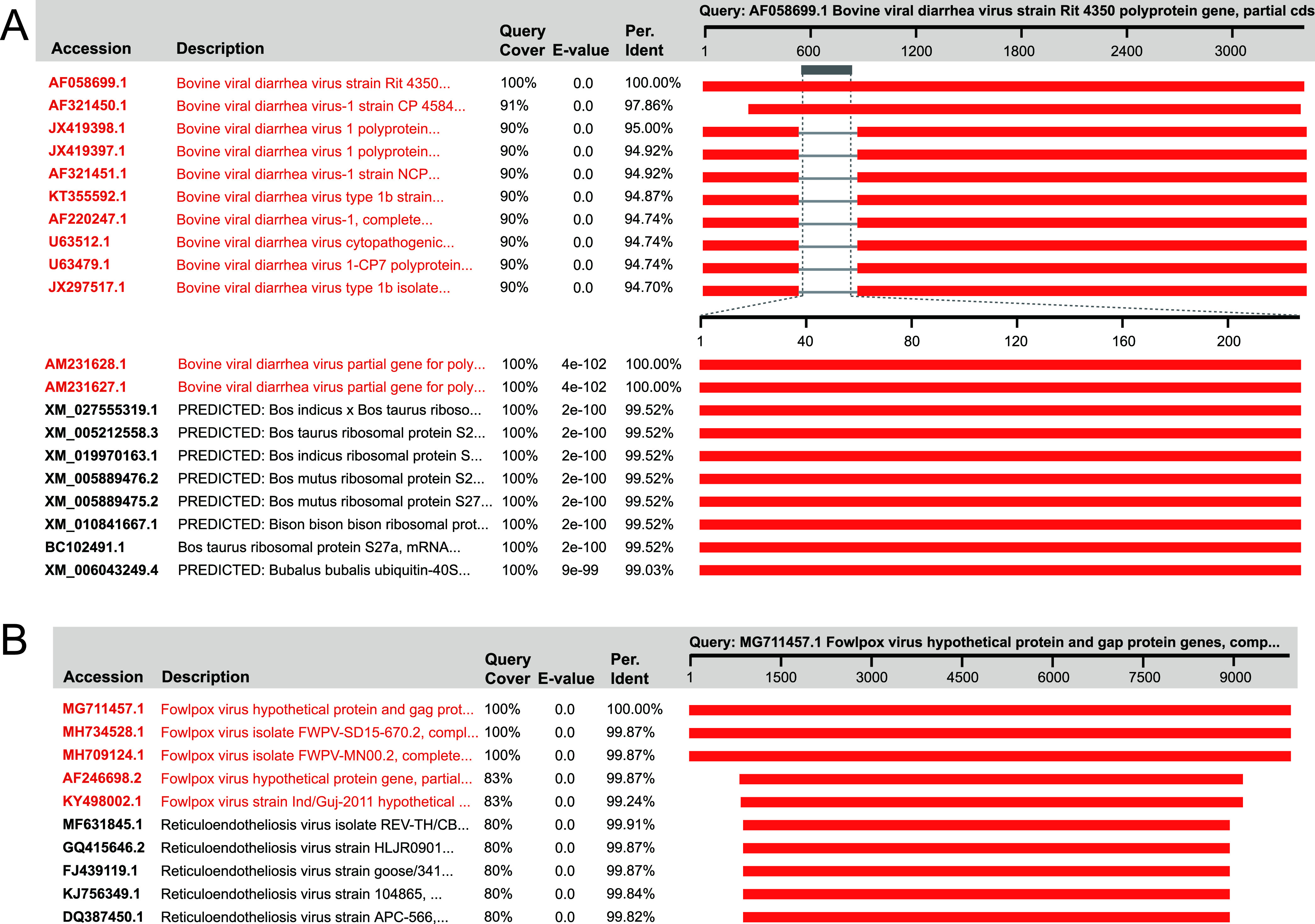
Identification of the na-PVSs of BVDV (A) and fowlpox virus (B) using blastn search. The blastn hits with close definition to the query are highlighted in red.

**(i) Natural occurrence.** Some na-PVSs are naturally acquired by viruses in the process of replication, which is essential for certain viruses to gain new features. Bovine viral diarrhea virus (BVDV) is a worldwide-distributed pathogen and can cause severe consequences to cattle and sheep (29). Almost all PVSs within the family *Flaviviridae* are inserts of bovine hybrid ribosomal S27a and ubiquitin sequences into the BVDV genomes (Fig. 2A). The in-frame insertion of the host sequence into the NS3 gene is essential for the virus to gain cytopathogenicity in cell culture (30). Hepatitis E virus (HEV) is hard to culture using cell systems; the integration of a short piece of human S17 ribosomal protein fragment into the hypervariable region of the HEV genome enables some variants to grow in HepG2/C3A cells (Data Set S1) (31). Besides host sequences, genomic fragments of other viral families can also integrate into some viral genomes, particularly during coinfection of multiple viruses. For some large DNA viruses, viral DNA replicates within the cellular nucleus or cytoplasm, providing an opportunity for a retrovirus to be integrated into a viral genome. Thus, avian retrovirus was shown to be integrated into the genome of Marek’s disease virus, an avian herpesvirus (Data Set S1) (32). We also detected reticuloendotheliosis virus sequences of various lengths, even nearly full length, integrated into the genomes of some fowlpox viruses (Fig. 2B), which likely enhanced the pathogenicity of the viruses (33, 34). Interfamily recombination can also occur in RNA viruses. A betacoronavirus detected in bats contained a unique gene at the 3′ end of its genome that most likely originated from the p10 gene of a bat orthoreovirus, a gene that can induce the formation of cell syncytia (35).

**FIG 3:**
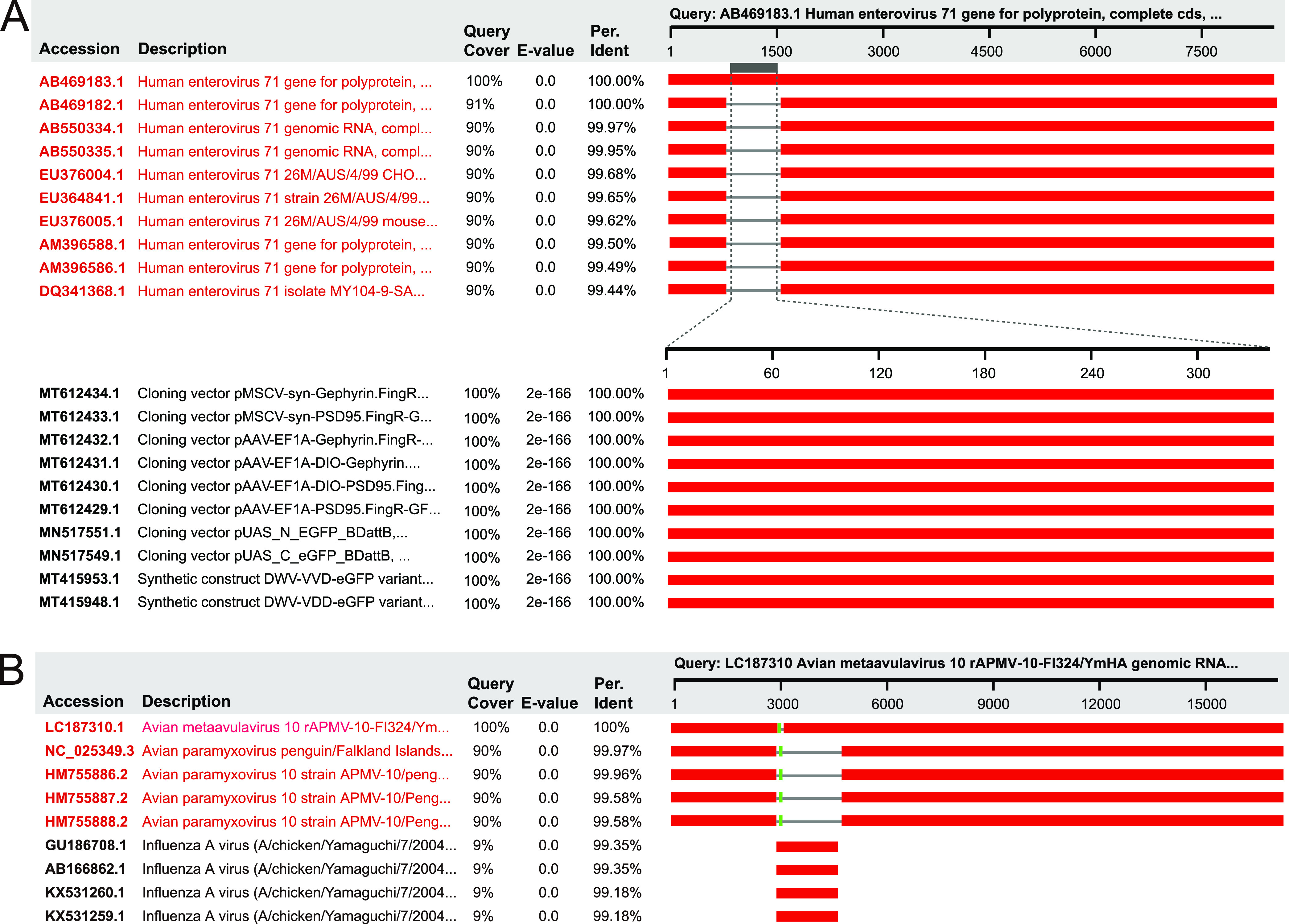
Identification of the ia-PVSs of human enterovirus 71 (A) and avian metaavulavirus (B) using blastn search. The blastn hits with close definition to the query are highlighted in red.

**(ii) Intentionally artificial occurrence.** Some viral genomes are intentionally engineered to contain foreign sequences that might derive from nonviral artifacts or viruses of other families, by which these engineered viruses provide important tools to study viral infection, deliver foreign proteins, or even combat diseases. We found that most vector PVSs (87.2%), a few misclassification PVSs (*n* = 3), and no host PVSs are intentional artifacts (Data Set S1). Among ia-PVSs with vector origins, green fluorescent proteins are very common (41.5%) (Fig. 3A and Data Set S1), and elements like neomycin phosphotransferase, mCherry, and firefly luciferase were also detected (Data Set S1). The three misclassified ia-PVSs are all associated with avian paramyxovirus (Data Set S1). These are actually artificial recombinants designed to serve as vaccine vectors to combat avian influenza (Fig. 3B) (36).

**FIG 4:**
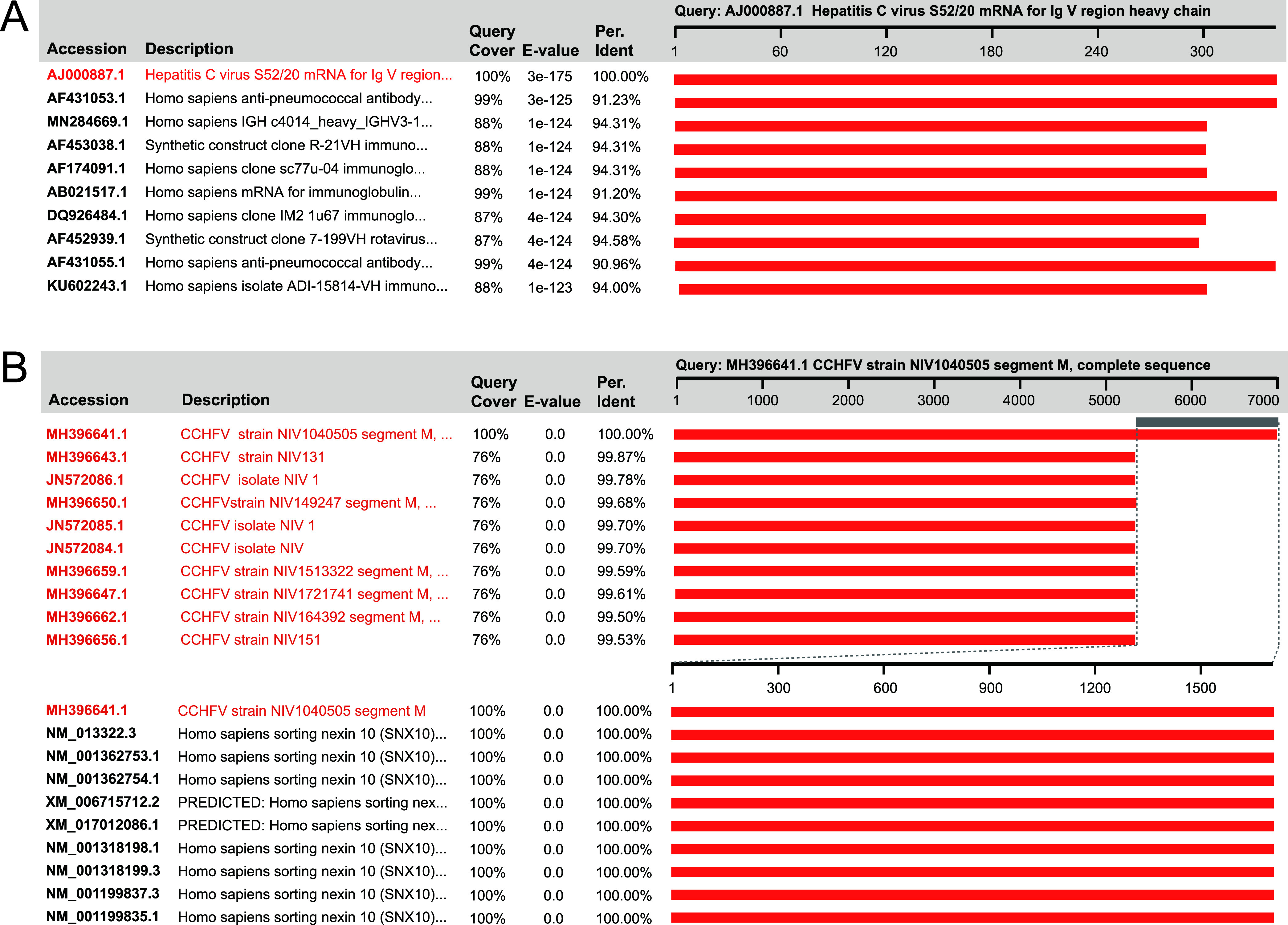
Identification of the ua-PVSs of hepatitis C virus (A) and CCHFV (B) using blastn search. The blastn hits with close definition to the query are highlighted in red.

**(iii) Unintentionally artificial occurrence.** The ua-PVSs are technically contaminating sequences but are unintentionally introduced into viral genomes. They are widely distributed in host, vector, laboratory component, and misclassification PVSs (Data Set S1). The host-origin ua-PVSs can be full-length sequences, e.g., a 399-bp-long human mRNA was erroneously defined as hepatitis C virus (Fig. 4A). Misassembly of HTS reads will result in chimeric ua-PVSs at the termini of a sequence, e.g., a 1,636-bp-long human sorting nexin 10 fragment was misassembled into the 3′ terminus of segment M of a Crimean-Congo hemorrhagic fever orthonairovirus (CCHFV) (Fig. 4B). As to ua-PVSs of vector origin, we found two short stealth virus sequences that are actually vector backbones (Data Set S1). Through multiple checks using viral metagenomes from different hosts, we found some viral reference sequences sharing >99% nt identities with viromic contigs of different host species. Viruses harbored by different host species are usually distinct from each other due to the genetic adaptation to a specific host species. If a virus is detected in multiple host species, it should be particularly noted whether it is the result of cross-species transmission or simply foreign contamination. Further verification showed that these reference sequences are all nonviral but are genomic fragments of bacteria (Data Set S1). For example, a bluetongue virus sequence (AY397620) frequently encountered in our viral metagenomic analyses is a Mycoplasma bovis chromosomal sequence (Data Set S1).

Cross-family misclassification can occur between eukaryotic viral families, even between eukaryotic and prokaryotic viral families. Three sequences wrapping circovirus-featured *rep* and *cap* genes should be classified into the family *Circoviridae* but are defined as dependoparvoviruses within the family *Parvoviridae* (Fig. S2). A 558-bp-long sapovirus sequence (AB212270) defined within the family *Caliciviridae* actually originated from a Salmonella phage (Data Set S1). If a viral sequence is highly novel with very low similarity to known references, it would be likely misclassified at the family level. A 4,047-bp-long sequence recovered from a bird metagenome was defined as *Parvoviridae* sp., but it had very few blastn hits in the nt database and several blastx hits against major capsid proteins of microviruses (Fig. S3A). Profile comparison showed that one of its proteins is perfectly matched to the capsid of microviruses with a probability of 100% (Fig. S3B). Accordingly, it should be classified as a bacteriophage rather than a parvovirus.

10.1128/msystems.00907-22.3 FIG S2 Identification of the misclassified anseriform dependoparvovirus using genomic structure prediction and blastn search. The blastn hits with close definition to the query are highlighted in red. Download FIG S2, TIF file, 0.3 MB. Copyright © 2022 Chen et al. 2022 Chen et al. https://creativecommons.org/licenses/by/4.0/ This content is distributed under the terms of the Creative Commons Attribution 4.0 International license.

10.1128/msystems.00907-22.4 FIG S3 Identification of the misclassified parvovirus using blastn/x searches (A) and Hidden Markov Model (HMM-HMM) comparison (B). Download FIG S3, TIF file, 4.1 MB. Copyright © 2022 Chen et al. 2022 Chen et al. https://creativecommons.org/licenses/by/4.0/ This content is distributed under the terms of the Creative Commons Attribution 4.0 International license.

Although these na- and ia-PVSs are essentially different from the foreign contaminants in other databases, they can, together with ua-PVSs, result in misleading conclusions in applications like virus detection, virome analysis, and taxonomic assignment. Therefore, we deleted or trimmed these PVSs from the PDS to build a clean version of the viral reference data set. However, the resulting data set is still redundant, with a high level of identical sequences. Thus, we removed the redundancy at 99% identity and 90% coverage, which downsized the nt and aa data sets for ~6 and ~3 times, respectively (Fig. S1).

### Functional enhancement of the data set.

We built this data set primarily for virome-based virus detection. However, the HTS-based viral metagenome is prone to be contaminated by LCD viral sequences and vector-derived viral functional cassettes (11, 12, 16, 37), which are also recorded in GenBank. This issue cannot be addressed by removing them from the reference database because those sequences would still be annotated by their genetically close relatives. Thus, we added these risk sequences into the data set by labeling them “LCD” or “Vector,” which can provide a warning; if a query shows extremely high similarity to them, it should be concerning whether the sample is contaminated by exogenous LCD sequences or vectors (12). Besides, attenuated viruses are widely used in human and animal vaccinations to combat infectious diseases. It is critical to distinguish them from field strains in clinical diagnosis. Therefore, we enhanced the data set function by the addition of labeled LCD (*n* = 155), viral functional cassette (*n* = 79), and vaccine (*n* = 40) sequences into the nt branch (Data Set S2). Vaccine sequences added here covered 15 attenuated viruses commonly used in humans and animals against mumps, equine infectious anemia, porcine epidemic diarrhea, etc. (Data Set S2). By such enhancement, the final eukaryotic viral reference data set (EVRD) was achieved, with the nt and aa sequences archived in the EVRD-nt and EVRD-aa branches, respectively. EVRD-nt has 558,638 sequences with an average length of 2,943 bp covering 117 families, while EVRD-aa catalogs 1,256,089 sequences from 115 families with an average length of 371 aa. At the time of manuscript preparation, we have updated the data set into version 2.0 with addition of 34,037 and 221,426 new sequences into EVRD-nt and EVRD-aa, respectively. These new sequences covered 111 families with the majority being retroviruses, picornaviruses, and reoviruses.

10.1128/msystems.00907-22.10 DATA SET S2 Details of the tagged LCD and vaccine sequences in EVRD-nt. Download Data Set S2, XLSX file, 0.02 MB. Copyright © 2022 Chen et al. 2022 Chen et al. https://creativecommons.org/licenses/by/4.0/ This content is distributed under the terms of the Creative Commons Attribution 4.0 International license.

### EVRD improves the accuracy and efficiency of virome analysis.

**FIG 5:**
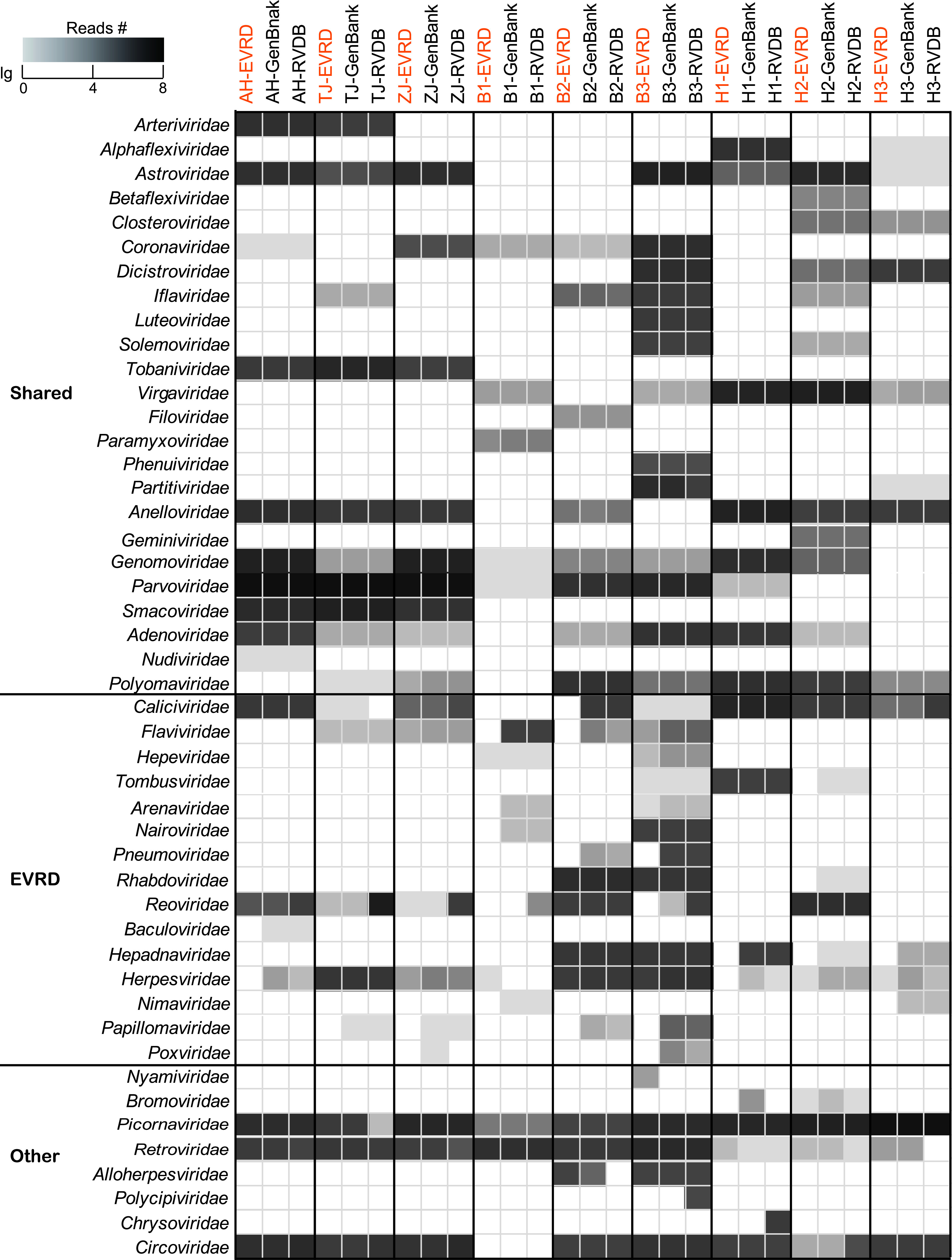
Comparison of the virus-like read numbers in nine viromic data sets annotated by blastn searches against EVRD-nt (highlighted in orange), GenBank, and RVDB-nt. Viral families are divided into categories of “Shared,” “EVRD,” and “Other,” corresponding to families that are coannotated by the three reference databases, not annotated by EVRD in certain data sets, and annotated by one or two reference databases in certain data sets, respectively.

The performance of EVRD in virome analysis was evaluated by comparison of its accuracy, coverage, and efficiency with GenBank (for nt) and UniProt (for aa) virus branches and RVDB (v21.0). RVDB is a reference viral database that provides a broad representation of different virus species from eukaryotes by including all viral, virus-like, and virus-related sequences (26). The detailed comparisons were summarized in Text S1. Briefly, using nine viral metagenomes of pigs, bats, and humans, the blastn annotation revealed that the majority (88.1%) of virus-like reads (VLRs) were coannotated by the three databases (Fig. 5), but EVRD massively reduced false annotations to calicivirus, BVDV, pneumovirus, etc. (Fig. 5). Annotation using contigs at the nt and aa levels also showed that EVRD prevented misannotation to calicivirus, reovirus, parvovirus, etc. (Fig. S4). These results indicated that EVRD does not sacrifice the detection spectrum of eukaryotic viruses but rather greatly improves the specificity and accuracy of viromic annotation (Text S1). Notably, we detected cocirculation of field and vaccine strains of porcine reproductive and respiratory syndrome viruses in viral metagenome AH (Text S1 and Fig. S5), which should be especially concerning, since new viruses could be generated through recombination between field viruses and vaccine strains. Viromic annotation is quite consuming of time and computing resources. A small-scale reference database can save the analytic time and minimize the computing resources. With an entry-level platform, analyses of reads or contigs at nt or aa levels using EVRD were 1.8 to 3.3 and 1.9 to 3.2 times faster than using GenBank/UniProt and RVDB, respectively (Fig. S6), indicating that EVRD is more time-saving and easier to configure.

10.1128/msystems.00907-22.5 FIG S4 Comparison of the viral contig numbers in the nine viromic data sets using blastn search against EVRD-nt, GenBank, and RVDB-nt. Download FIG S4, TIF file, 0.7 MB. Copyright © 2022 Chen et al. 2022 Chen et al. https://creativecommons.org/licenses/by/4.0/ This content is distributed under the terms of the Creative Commons Attribution 4.0 International license.

10.1128/msystems.00907-22.6 FIG S5 Identification of virus-like reads (VLRs). (A) VLRs were annotated using different databases with read numbers shown in each subset. (B) The annotations of VLRs in the six subsets were improved using a length cutoff of 100 (orange bars); some VLRs could be annotated using the other databases with a length of <100 (yellow bars), but there were still some VLRs (gray bars, labeled using Ex) unable to be annotated by the other databases even when length was loosened to <100. (C) The Ex VLRs in subset E∩G were all related to Osugoroshi viruses within the family *Partitiviridae*. (D) The Ex VLRs in subsets G and G∩R were all associated with PVSs. (E) The Ex VLRs in subset R were predominantly annotated by RVDB-exclusive viral metagenomes. (F) The porcine reproductive and respiratory syndrome virus (PRRSV) VLRs in data set AH belonged to vaccine and field strains based on the annotation using EVRD-nt. Download FIG S5, TIF file, 1.0 MB. Copyright © 2022 Chen et al. 2022 Chen et al. https://creativecommons.org/licenses/by/4.0/ This content is distributed under the terms of the Creative Commons Attribution 4.0 International license.

10.1128/msystems.00907-22.7 FIG S6 Time consumption of the three reference databases in blastn and blastx analyses of reads and contigs of the nine viromic data sets on an entry-level computing platform. Download FIG S6, TIF file, 0.3 MB. Copyright © 2022 Chen et al. 2022 Chen et al. https://creativecommons.org/licenses/by/4.0/ This content is distributed under the terms of the Creative Commons Attribution 4.0 International license.

## DISCUSSION

A high-quality database is critical in bioinformatic analyses in biology, biotechnology, and medicine. To maintain the quality of the database, the National Center for Biotechnology Information applies several protocols to screen submitted sequences for contamination, such as using the program VecScreen and cross-checking using BLAST search (7, 38). However, such measures are insufficient to provide adequate quality assurance. The original submitters should carefully check the quality of the sequence(s) before they submit it. As to viral sequences, many aspects can be considered to improve their quality. First, the viral genome should be thoroughly annotated to indicate all foreign sequences that occur naturally or are introduced intentionally, so that users can understand that they are related to evolutionary processes or biomedical use, as well as take note of their heterogeneity. Second, although HTS-based viral metagenomics has greatly promoted the rate of new virus discovery, there is an inevitable issue in which viral genomes can be misassembled to include a foreign insert(s) and a misplaced gene(s) (39). Thus, those new sequences should be validated by multiple means, such as PCR amplification and Sanger sequencing. Third, the emerging profile-based and machine learning-based virome annotation algorithms allow us to explore those remote viruses that show very limited similarities to known references (40, 41), but it would be difficult to accurately assign their taxonomic lineages. In such cases, classification based on multiple criteria such as virus hallmark gene annotation and genomic organization is helpful. Virus hallmark genes encode very conserved proteins that are usually shared by broad ranges of viruses, which can be used as indicators in high-rank taxonomic classification (42), while viruses within a low-rank taxon have common genomic organization, which helps to determine taxa below family level. Last but not least, users should be aware of these problems caused by PVSs when using databases and need to realize that a careful review of bioinformatic results is necessary for a reliable conclusion.

In other databases, those foreign sequences are considered contaminating ones, among which human sequences contribute a substantial part (6–10). It is not surprising that host sequences are the predominant PVSs because host cells provide the obligatory venue for viruses to replicate. However, unlike organisms in other kingdoms, viruses are characterized by unique and diverse replication strategies, with some capable of exchanging genomic fragments with their hosts (43), and they are also easily genetically modified to contain foreign DNA for various biomedical purposes. Thus, these host PVSs cannot be arbitrarily considered foreign contaminants, since some of them are actually natural products or intentional artifacts, which explains why the foreign sequences in viral databases are much more complicated and more difficult to differentiate than those in databases of other organisms (7). However, no matter how they originate, these PVSs can cause a variety of problems in downstream studies, particularly for evolutionary analysis and genome annotation.

EVRD is maintained in FASTA format with informative identifiers. It can be easily localized and configured according to any similarity-based sequence analytical software, such as BLAST suite for sequence search or MMSeqs2 for sequence cluster. EVRD provides a valuable resource for, but not limited to, these application scenarios below. Accurate determination of causative agents is a priority in clinical diagnosis of EIDs. EVRD can be widely used in virome-based viral disease diagnosis since it avoids those false positives caused by misannotation of PVSs. EVRD can also improve the taxonomic classification of viral sequences in the assessment of virus diversity (39). In such analysis, viral contigs need to be clustered with reference sequences, but these PVSs, specifically the cross-family misclassified ones, will disturb the boundary of virus clusters and even result in incorrect taxonomic classification. In addition, multiple sequence alignments (MSAs) are prone to being corrupted by PVSs. The refined EVRD sequences can help build high-quality MSAs that are the basis of profiles of clustered sequences, thus favoring the exploration of remote viruses.

Besides utilizing a high-quality reference database, other measures can be considered to improve the reliability of viromics-based virus detection. According to different purposes, we should choose a reasonable bioinformatic pipeline. Annotation using reads provides richer information than using contigs and hence is helpful to capture ultralow-abundance viruses in virus detection (44, 45). But sequence completeness is a priority in viral ecology, so assembly is preferentially performed before annotation (39). The criterion used to determine a viral sequence has a substantial impact on virome annotation (see Text S1 in the supplemental material). Regarding read, the criterion is mainly based on E value, but the alignment length is also an important factor to help increase the confidence level of annotation. Besides E value and length, the requirement of a minimum gene number has been widely considered in contig annotation (39). The quality of assemblies should be seriously considered in contig annotation. There are many means to improve the assembly quality, such as choosing suitable software (46), employing a rational sample treatment protocol (47), and reducing the bias induced by random amplification (48). A classification of host and other microorganism reads prior to *de novo* assembly could help reduce chimeric contigs (47). Besides, a final check provides an additional guarantee for high-quality annotation (49), e.g., host contamination should be checked and eliminated as much as possible. Prokaryotic host contamination can be determined using CheckV, but a different strategy is needed to deal with eukaryotic host contamination (49, 50).

In conclusion, we detected these PVSs hidden in the largest viral nt and aa databases using a systematic scrutiny pipeline, followed by careful identification of their types and origins, which not only refreshes our knowledge of the occurrence of PVSs but also provides a clean version of the eukaryotic viral reference data set. The scrutiny pipeline is designed for eukaryotic viruses but is not suitable for prokaryotic ones. Bacteriophages, specifically lysogenic ones, frequently exchange their genomic elements with their hosts, making it very difficult to differentiate foreign contamination from genomic integration. But it is feasible to identify host contamination in phage genomes by creating and querying a distinctive bacterial gene set. Besides, it is important to note that we excluded those large DNA viruses infecting eukaryotic microorganisms, due to their extraordinarily large and complicated genomes and the lack of evidence that they cause diseases in vertebrates (51–53). Though we have deleted hundreds of PVSs of vertebrate-infecting large DNA viruses from families like *Herpesviridae* and *Poxviridae*, there are still some ambiguous sequences that can be treated as host PVSs if using loose criteria. Thus, annotations to these viruses using EVRD should still be verified with caution. Additionally, these tagged warning sequences in EVRD are very useful, but they are partial and represent only the sequences we have searched so far. We will keep the database updated with new advances in this regard.

## MATERIALS AND METHODS

### Scrutiny pipeline for nucleotide sequences.

**(i) Preliminary filtration.** We first generated the taxonomic lineages of all sequences and then removed those lineages infecting bacteria, archaea, fungi, and eukaryotic microorganisms using the relationship of virus and host recorded in the ViralZone database (54). In addition, as there are a large number of sequences that cannot be assigned to a complete lineage, we searched their definition using keywords and removed the sequences related to prokaryotic and environmental viruses and metagenomes, such as bacteriophage/phage, environmental and uncultured viruses, and ameba viruses. The gbvrl division also deposits a large number of sequences with lengths of ≤200 bp, which are highly similar to the longer ones and contribute little to genomic analysis; hence, they were also removed.

**(ii) Host genome scrutiny.** In this part, fragments of host genomes in the remaining sequences of the PDS were detected. Genomic assemblies of human (*n* = 1), pig (*n* = 1), bat (*n* = 7), rodent (*n* = 2), arthropod (*n* = 11), cattle (*n* = 1), dog (*n* = 1), cat (*n* = 1), sheep (*n* = 1), chicken (*n* = 1), and mallard (*n* = 1) were used to perform a blastn search against these sequences with a maximum of 1,000 subjects to show alignments (length of ≥150 and identity of ≥85%). Retroviruses can infect almost all vertebrates, resulting in thousands of loci of retroviral sequences in vertebrate genomes (55). Here, we did not challenge the known ambiguity of retroviruses, and hence, hits to retroviruses were not considered. The aligned sequences of the subject were extracted and subjected to blastn search against the nt database to further validate their identities. The top 100 hits of each sequence were kept, and if ≥80% of these hits were annotated as nonviral, the aligned sequence was considered problematic. The original sequence was removed from the PDS if its problematic part comprised ≥80% of its length or was trimmed by deleting the problematic parts. Such a threshold was also applied to the following treatments. Such treatment was iterated until no host genomic fragments were found.

**(iii) Vector sequence scrutiny.** To detect PVSs derived from backbones or functional cassettes of vectors, the UniVec database and sequences of ≥1,000 bp under the GenBank taxonomy of vectors (uid: 29278) were downloaded. As vectors have many functional cassettes originating from viruses, such as simian virus 40 (SV40) and cytomegalovirus (CMV) promoters and retroviral *gag* and *pol* elements, these vector-originating PVSs in the PDS were carefully detected and examined using the following procedure to prevent any erroneous deletions of genuine viral sequences. We generated a nonviral protein core (NVPC) that consists of nonviral expression elements (*n* = 13,287) originating in vectors (see Text S1 in the supplemental material). Sequences in the PDS were subjected to a blastx search against the NVPC using Diamond with those showing ≥99% similarity over an alignment of ≥60 aa to subjects being pruned. In addition, UniVec was used to identify adapters, linkers, and primers that are often used to clone sequences. The remaining sequences in the PDS were further scrutinized using the procedure introduced under “(ii) Host genome scrutiny” with the same criteria. Briefly, these downloaded vector sequences were used as queries to search for possible subjects in the PDS using blastn. Hits in the PDS were further validated by blastn searches against the nt database. After the removal of those vector-originating sequences, the rest of the PDS was reexamined until no vector sequences existed.

**(iv) Misclassification scrutiny.** Erroneous taxonomic annotation of viral sequences was detected by all-against-all blastn searches with a maximum of 1,000 subjects to show alignments. We found that there are a large number of sequences with correct taxonomic annotation showing intrafamily cross-species/genus blastn hits, such as *Betacoronavirus*/*Gammacoronavirus* within the family *Coronaviridae*, *Tetraparvovirus*/*Protoparvovirus* of the family *Parvoviridae*, and *Circovirus*/*Cyclovirus* within the family *Circoviridae*, which were likely ascribed to high similarity between species/genera. Hence, we inspected annotation at the family level. Here, we defined that a blastn hit is significant if its E value is ≤1e−50 and length is ≥500. If the proportion of alignments that were generated by a query against subjects of a different family to all alignments of the query was ≥80%, the query was considered to be possibly misclassified and was further subjected to genomic organization identification, in which if the genomic organization of the query was not typical of the features its defined taxonomic lineage should have had, the query was truly misclassified and was removed from the PDS. During treatment, we noted that some sequences had a few alignments (usually ≤10) that showed ≤80% similarities with subjects of different families, and we kept their original annotations since there are not enough references in GenBank to determine their true taxonomic lineages.

**(v) LCD sequence detection.** Previous studies showed that some viral contaminant sequences are highly prevalent in cross-host HTS-based viromic data, which might be linked to biological or synthetic products (11, 12, 16). To examine whether cross-host sequences exist in the database, the remaining sequences in the PDS were subjected to a cross-check of viral metagenomes. A total of 15 viromic raw data sets covering humans, bats, ticks, rodents, bovine, pigs, and avians were downloaded from the SRA and respectively *de novo* assembled. Contigs of ≥1,000 bp were subjected to blastn search against the PDS with a maximum of 1,000 subjects to show alignments. If a subject was matched by contigs from viromic data sets of two or more different hosts with alignment of ≥150 bp and identity of ≥80%, it was classified as a suspicious sequence and further validated by blastn search against the nt database. If a suspicious sequence was annotated to nonviral species by blastn searches against the nt database, it was considered a truly exogenous contaminating sequence and removed from the PDS. However, if a suspicious sequence was still annotated to virus and shared 99% nt identities with viromic contigs of two or more different hosts, it was considered a truly viral sequence but one probably originating from laboratory-component-derived viral sequence contamination and hence was retained in the PDS but was tagged as LCD. The remaining suspicious sequences were passed and kept in the PDS.

### Scrutiny pipeline for viral protein sequences.

The protein sequences were retrieved from the UniProt virus division. We first checked their representativeness. In case there were any coding regions not annotated by the original submitters, all proteins of the PDS nt sequences prior to filtration were *de novo* predicted using Prodigal v2.6.3 with meta mode. Proteins of ≥50 aa were blastp searched against the UniProt viral division (E value of ≤1e−10 and sequence identity of ≥90), and the results revealed that the UniProt viral division has high representativeness with 99.6% consistency with the prediction of GenBank viruses. Therefore, we simply used UniProt sequences to screen PVSs as described for scrutiny of nt sequences with minor modification (Text S1). Although these nt and aa PVSs were independently detected, we compared the counterparts of aa PVSs in their nt sequences with these nt PVSs to further confirm that these sequences are problematic.

### EVRD finalization.

After the above scrutiny, the sequences in the PDS were still very redundant, and hence, a deredundance procedure was applied to downsize the PDS. Clustering of viral nt and aa sequences was performed using MMseq2 (56) with a sequence similarity threshold of 0.99 and 90% coverage of the short sequence. The LCD sequences reported previously (12, 16) and identified here were tagged using “LCD” and added into the PDS as risk sequences. To distinguish viral functional cassettes from true virus sequences, the sequences corresponding to the regulatory classes of promoter, terminator, and enhancer and/or with notes containing the word “virus” were extracted from vectors and subjected to blastn search against the nonredundant PDS. The vector sequences verified to be viral were dereplicated and also added into the PDS with tags of “Vector.” In addition, we collected vaccine strains commonly used in humans and other animals by searching publications or by personal communication (Data Set S2). These vaccine nt sequences were also tagged using “Vaccine” and added into the PDS.

### Performance evaluation of EVRD.

We evaluated the performance of EVRD by comparison with gbvrl (for nt), UniProt (for aa), and RVDB at the read and contig levels. A total of nine viral metagenomic raw data sets (Table S1) were subjected to host genome removal using Bowtie2 (v2.4.1) with sensitive mode and then taxonomically classified using Kraken2 (v2.0.9-beta) to remove bacterial, archaeal, and fungal reads. First, the unassigned reads were directly blastn (E value of ≤10e−5 and length of ≥120) and blastx (E value of ≤10e−5 and length of ≥40) searched against these databases. Then, they were *de novo* assembled using megahit (v1.2.9). Contigs of ≥1,000 bp were retained for blastn (v2.10.0) and Diamond blastx (v0.9.35) search against the nt and aa reference databases, respectively. The blastn hit of a contig to a subject with one alignment with an E value of ≤10e−10 and a length of ≥450 or two or more alignments with an E value of ≤10e−5 and a length of ≥150 was considered positive, and the blastx hit to a subject was recognized as positive if it had one alignment with an E value of ≤10e−10 and a length of ≥150 or two or more alignments with an E value of ≤10e−5 and a length of ≥50. The positive reads and contigs were further verified by blastn/x search against nt/nr databases (18). All blast searches were performed using 12 × 86_64 central processing units (CPUs) of an Inter Xeon Gold 2.660-GHz processor. To detect if a data set contained warning sequences, we defined a rigorous cutoff, i.e., if a sequence hit a tagged subject with an identity of ≥99% and coverage of the query of ≥90% by blastn search against EVRD-nt, it was considered to be a warning sequence.

10.1128/msystems.00907-22.8 TABLE S1 Details of the nine viromic data sets used to evaluate the performance of the three reference databases. Data sets H1, H2, and H3 had many runs, and they (marked A in H2 and C in H3) were respectively merged in the analysis. Download Table S1, XLSX file, 0.01 MB. Copyright © 2022 Chen et al. 2022 Chen et al. https://creativecommons.org/licenses/by/4.0/ This content is distributed under the terms of the Creative Commons Attribution 4.0 International license.

### Data availability.

All data used here were downloaded from relevant databases. The accession numbers of viral metagenomic raw data and genome identifiers (IDs) are summarized in Fig. S1 and Text S1. The key intermediate data (NVPC) and essential codes are available from http://github.com/BH-Lab/EVRD. The code for viromic analysis using EVRD has been published elsewhere (18). EVRD reported here is scheduled to annually update, and the current version is v2.0, which is freely accessible at http://cvri.caas.cn/kxyj/yjfx/bfbd/EVRD/index.htm.

## References

[1] Jones KE, Patel NG, Levy MA, Storeygard A, Balk D, Gittleman JL, Daszak P. 2008. Global trends in emerging infectious diseases. Nature 451:990–993. doi:10.1038/nature06536. 18288193 PMC5960580

[2] Zhu N, Zhang D, Wang W, Li X, Yang B, Song J, Zhao X, Huang B, Shi W, Lu R, Niu P, Zhan F, Ma X, Wang D, Xu W, Wu G, Gao GF, Tan W, China Novel Coronavirus Investigating and Research Team. 2020. A novel coronavirus from patients with pneumonia in China, 2019. N Engl J Med 382:727–733. doi:10.1056/NEJMoa2001017. 31978945 PMC7092803

[3] Venkatesan P. 2022. Global monkeypox outbreak. Lancet Infect Dis 22:950. doi:10.1016/S1473-3099(22)00379-6. 35752185 PMC9533939

[4] Wilson MR, Sample HA, Zorn KC, Arevalo S, Yu G, Neuhaus J, Federman S, Stryke D, Briggs B, Langelier C, Berger A, Douglas V, Josephson SA, Chow FC, Fulton BD, DeRisi JL, Gelfand JM, Naccache SN, Bender J, Dien Bard J, Murkey J, Carlson M, Vespa PM, Vijayan T, Allyn PR, Campeau S, Humphries RM, Klausner JD, Ganzon CD, Memar F, Ocampo NA, Zimmermann LL, Cohen SH, Polage CR, DeBiasi RL, Haller B, Dallas R, Maron G, Hayden R, Messacar K, Dominguez SR, Miller S, Chiu CY. 2019. Clinical metagenomic sequencing for diagnosis of meningitis and encephalitis. N Engl J Med 380:2327–2340. doi:10.1056/NEJMoa1803396. 31189036 PMC6764751

[5] Zhou P, Yang X-L, Wang X-G, Hu B, Zhang L, Zhang W, Si H-R, Zhu Y, Li B, Huang C-L, Chen H-D, Chen J, Luo Y, Guo H, Jiang R-D, Liu M-Q, Chen Y, Shen X-R, Wang X, Zheng X-S, Zhao K, Chen Q-J, Deng F, Liu L-L, Yan B, Zhan F-X, Wang Y-Y, Xiao G-F, Shi Z-L. 2020. A pneumonia outbreak associated with a new coronavirus of probable bat origin. Nature 579:270–273. doi:10.1038/s41586-020-2012-7. 32015507 PMC7095418

[6] Breitwieser FP, Pertea M, Zimin AV, Salzberg SL. 2019. Human contamination in bacterial genomes has created thousands of spurious proteins. Genome Res 29:954–960. doi:10.1101/gr.245373.118. 31064768 PMC6581058

[7] Steinegger M, Salzberg SL. 2020. Terminating contamination: large-scale search identifies more than 2,000,000 contaminated entries in GenBank. Genome Biol 21:115. doi:10.1186/s13059-020-02023-1. 32398145 PMC7218494

[8] Merchant S, Wood DE, Salzberg SL. 2014. Unexpected cross-species contamination in genome sequencing projects. PeerJ 2:e675. doi:10.7717/peerj.675. 25426337 PMC4243333

[9] Lu J, Salzberg SL. 2018. Removing contaminants from databases of draft genomes. PLoS Comput Biol 14:e1006277. doi:10.1371/journal.pcbi.1006277. 29939994 PMC6034898

[10] Longo MS, O’Neill MJ, O’Neill RJ. 2011. Abundant human DNA contamination identified in non-primate genome databases. PLoS One 6:e16410. doi:10.1371/journal.pone.0016410. 21358816 PMC3040168

[11] Rosseel T, Pardon B, De Clercq K, Ozhelvaci O, Van Borm S. 2014. False-positive results in metagenomic virus discovery: a strong case for follow-up diagnosis. Transbound Emerg Dis 61:293–299. doi:10.1111/tbed.12251. 24912559

[12] Asplund M, Kjartansdóttir KR, Mollerup S, Vinner L, Fridholm H, Herrera JAR, Friis-Nielsen J, Hansen TA, Jensen RH, Nielsen IB, Richter SR, Rey-Iglesia A, Matey-Hernandez ML, Alquezar-Planas DE, Olsen PVS, Sicheritz-Pontén T, Willerslev E, Lund O, Brunak S, Mourier T, Nielsen LP, Izarzugaza JMG, Hansen AJ. 2019. Contaminating viral sequences in high-throughput sequencing viromics: a linkage study of 700 sequencing libraries. Clin Microbiol Infect 25:1277–1285. doi:10.1016/j.cmi.2019.04.028. 31059795

[13] Campbell SJ, Ashley W, Gil-Fernandez M, Newsome TM, Di Giallonardo F, Ortiz-Baez AS, Mahar JE, Towerton AL, Gillings M, Holmes EC, Carthey AJR, Geoghegan JL. 2020. Red fox viromes in urban and rural landscapes. Virus Evol 6:veaa065. doi:10.1093/ve/veaa065. 33365150 PMC7744383

[14] Šimić I, Zorec TM, Lojkić I, Krešić N, Poljak M, Cliquet F, Picard-Meyer E, Wasniewski M, Zrnčić V, Ćukušić A, Bedeković T. 2020. Viral metagenomic profiling of Croatian bat population reveals sample and habitat dependent diversity. Viruses 12:891. doi:10.3390/v12080891. 32824037 PMC7472731

[15] Galindo I, Alonso C. 2017. African swine fever virus: a review. Viruses 9:103. doi:10.3390/v9050103. 28489063 PMC5454416

[16] Naccache SN, Greninger AL, Lee D, Coffey LL, Phan T, Rein-Weston A, Aronsohn A, Hackett J, Delwart EL, Chiu CY. 2013. The perils of pathogen discovery: origin of a novel parvovirus-like hybrid genome traced to nucleic acid extraction spin columns. J Virol 87:11966–11977. doi:10.1128/JVI.02323-13. 24027301 PMC3807889

[17] Knox K, Carrigan D, Simmons G, Teque F, Zhou Y, Hackett J, Qiu X, Luk K, Schochetman G, Knox A, Kogelnik A, Levy J. 2011. No evidence of murine-like gammaretroviruses in CFS patients previously identified as XMRV-infected. Science 333:94–97. doi:10.1126/science.1204963. 21628393

[18] He B, Gong W, Yan X, Zhao Z, Yang L, Tan Z, Xu L, Zhu A, Zhang J, Rao J, Yu X, Jiang J, Lu Z, Zhang Y, Wu J, Li Y, Shi Y, Jiang Q, Chen X, Tu C. 2021. Viral metagenome-based precision surveillance of pig population at large scale reveals viromic signatures of sample types and influence of farming management on pig virome. mSystems 6:e00420-21. doi:10.1128/mSystems.00420-21. 34100634 PMC8269232

[19] He B, Li Z, Yang F, Zheng J, Feng Y, Guo H, Li Y, Wang Y, Su N, Zhang F, Fan Q, Tu C. 2013. Virome profiling of bats from Myanmar by metagenomic analysis of tissue samples reveals more novel mammalian viruses. PLoS One 8:e61950. doi:10.1371/journal.pone.0061950. 23630620 PMC3632529

[20] Hu D, Zhu C, Wang Y, Ai L, Yang L, Ye F, Ding C, Chen J, He B, Zhu J, Qian H, Xu W, Feng Y, Tan W, Wang C. 2017. Virome analysis for identification of novel mammalian viruses in bats from Southeast China. Sci Rep 7:10917. doi:10.1038/s41598-017-11384-w. 28883450 PMC5589946

[21] Meng F, Ding M, Tan Z, Zhao Z, Xu L, Wu J, He B, Tu C. 2019. Virome analysis of tick-borne viruses in Heilongjiang province, China. Ticks Tick Borne Dis 10:412–420. doi:10.1016/j.ttbdis.2018.12.002. 30583876

[22] Tan Z, Yu H, Xu L, Zhao Z, Zhang P, Qu Y, He B, Tu C. 2019. Virome profiling of rodents in Xinjiang Uygur Autonomous Region, China: isolation and characterization of a new strain of Wenzhou virus. Virology 529:122–134. doi:10.1016/j.virol.2019.01.010. 30685659

[23] He W-T, Hou X, Zhao J, Sun J, He H, Si W, Wang J, Jiang Z, Yan Z, Xing G, Lu M, Suchard MA, Ji X, Gong W, He B, Li J, Lemey P, Guo D, Tu C, Holmes EC, Shi M, Su S. 2022. Virome characterization of game animals in China reveals a spectrum of emerging pathogens. Cell 185:1117–1129.e8. doi:10.1016/j.cell.2022.02.014. 35298912 PMC9942426

[24] Mukherjee S, Huntemann M, Ivanova N, Kyrpides NC, Pati A. 2015. Large-scale contamination of microbial isolate genomes by Illumina PhiX control. Stand Genomic Sci 10:18. doi:10.1186/1944-3277-10-18. 26203331 PMC4511556

[25] Pickett BE, Sadat EL, Zhang Y, Noronha JM, Squires RB, Hunt V, Liu M, Kumar S, Zaremba S, Gu Z, Zhou L, Larson CN, Dietrich J, Klem EB, Scheuermann RH. 2012. ViPR: an open bioinformatics database and analysis resource for virology research. Nucleic Acids Res 40:D593–D598. doi:10.1093/nar/gkr859. 22006842 PMC3245011

[26] Goodacre N, Aljanahi A, Nandakumar S, Mikailov M, Khan AS. 2018. A reference viral database (RVDB) to enhance bioinformatics analysis of high-throughput sequencing for novel virus detection. mSphere 3:e00069-18. doi:10.1128/mSphereDirect.00069-18. 29564396 PMC5853486

[27] Brister JR, Ako-Adjei D, Bao Y, Blinkova O. 2015. NCBI viral genomes resource. Nucleic Acids Res 43:D571–D577. doi:10.1093/nar/gku1207. 25428358 PMC4383986

[28] UniProt Consortium. 2021. UniProt: the universal protein knowledgebase in 2021. Nucleic Acids Res 49:D480–D489. doi:10.1093/nar/gkaa1100. 33237286 PMC7778908

[29] Lanyon SR, Hill FI, Reichel MP, Brownlie J. 2014. Bovine viral diarrhoea: pathogenesis and diagnosis. Vet J 199:201–209. doi:10.1016/j.tvjl.2013.07.024. 24053990

[30] Becher P, Orlich M, Thiel H-J. 1998. Ribosomal S27a coding sequences upstream of ubiquitin coding sequences in the genome of a pestivirus. J Virol 72:8697–8704. doi:10.1128/JVI.72.11.8697-8704.1998. 9765411 PMC110283

[31] Shukla P, Nguyen HT, Faulk K, Mather K, Torian U, Engle RE, Emerson SU. 2012. Adaptation of a genotype 3 hepatitis E virus to efficient growth in cell culture depends on an inserted human gene segment acquired by recombination. J Virol 86:5697–5707. doi:10.1128/JVI.00146-12. 22398290 PMC3347312

[32] Isfort RJ, Qian Z, Jones D, Silva RF, Witter R, Kung H-J. 1994. Integration of multiple chicken retroviruses into multiple chicken herpesviruses: herpesviral gD as a common target of integration. Virology 203:125–133. doi:10.1006/viro.1994.1462. 8030268

[33] Hertig C, Coupar BEH, Gould AR, Boyle DB. 1997. Field and vaccine strains of fowlpox virus carry integrated sequences from the avian retrovirus, reticuloendotheliosis virus. Virology 235:367–376. doi:10.1006/viro.1997.8691. 9281517

[34] Zhao K, He W, Xie S, Song D, Lu H, Pan W, Zhou P, Liu W, Lu R, Zhou J, Gao F. 2014. Highly pathogenic fowlpox virus in cutaneously infected chickens, China. Emerg Infect Dis 20:1208–1210. doi:10.3201/eid2007.131118. 24963887 PMC4073872

[35] Huang C, Liu WJ, Xu W, Jin T, Zhao Y, Song J, Shi Y, Ji W, Jia H, Zhou Y, Wen H, Zhao H, Liu H, Li H, Wang Q, Wu Y, Wang L, Liu D, Liu G, Yu H, Holmes EC, Lu L, Gao GF. 2016. A bat-derived putative cross-family recombinant coronavirus with a reovirus gene. PLoS Pathog 12:e1005883. doi:10.1371/journal.ppat.1005883. 27676249 PMC5038965

[36] Tsunekuni R, Hikono H, Tanikawa T, Kurata R, Nakaya T, Saito T. 2017. Recombinant avian paramyxovirus serotypes 2, 6, and 10 as vaccine vectors for highly pathogenic avian influenza in chickens with antibodies against Newcastle disease virus. Avian Dis 61:296–306. doi:10.1637/11512-100616-RegR1. 28957006

[37] Jurasz H, Pawłowski T, Perlejewski K. 2021. Contamination issue in viral metagenomics: problems, solutions, and clinical perspectives. Front Microbiol 12:745076. doi:10.3389/fmicb.2021.745076. 34745046 PMC8564396

[38] Schäffer AA, Nawrocki EP, Choi Y, Kitts PA, Karsch-Mizrachi I, McVeigh R. 2018. VecScreen_plus_taxonomy: imposing a tax(onomy) increase on vector contamination screening. Bioinformatics 34:755–759. doi:10.1093/bioinformatics/btx669. 29069347 PMC6030928

[39] Roux S, Adriaenssens EM, Dutilh BE, Koonin EV, Kropinski AM, Krupovic M, Kuhn JH, Lavigne R, Brister JR, Varsani A, Amid C, Aziz RK, Bordenstein SR, Bork P, Breitbart M, Cochrane GR, Daly RA, Desnues C, Duhaime MB, Emerson JB, Enault F, Fuhrman JA, Hingamp P, Hugenholtz P, Hurwitz BL, Ivanova NN, Labonté JM, Lee K-B, Malmstrom RR, Martinez-Garcia M, Mizrachi IK, Ogata H, Páez-Espino D, Petit M-A, Putonti C, Rattei T, Reyes A, Rodriguez-Valera F, Rosario K, Schriml L, Schulz F, Steward GF, Sullivan MB, Sunagawa S, Suttle CA, Temperton B, Tringe SG, Thurber RV, Webster NS, Whiteson KL, Wilhelm SW, Wommack KE, Woyke T, Wrighton KC, Yilmaz P, Yoshida T, Young MJ, Yutin N, Allen LZ, Kyrpides NC, Eloe-Fadrosh EA. 2019. Minimum information about an uncultivated virus genome (MIUViG). Nat Biotechnol 37:29–37. doi:10.1038/nbt.4306. 30556814 PMC6871006

[40] Ren J, Song K, Deng C, Ahlgren NA, Fuhrman JA, Li Y, Xie X, Poplin R, Sun F. 2020. Identifying viruses from metagenomic data using deep learning. Quant Biol 8:64–77. doi:10.1007/s40484-019-0187-4. 34084563 PMC8172088

[41] Guo J, Bolduc B, Zayed AA, Varsani A, Dominguez-Huerta G, Delmont TO, Pratama AA, Gazitúa MC, Vik D, Sullivan MB, Roux S. 2021. VirSorter2: a multi-classifier, expert-guided approach to detect diverse DNA and RNA viruses. Microbiome 9:37. doi:10.1186/s40168-020-00990-y. 33522966 PMC7852108

[42] Koonin EV, Dolja VV, Krupovic M, Varsani A, Wolf YI, Yutin N, Zerbini FM, Kuhn JH. 2020. Global organization and proposed megataxonomy of the virus world. Microbiol Mol Biol Rev 84:e00061-19. doi:10.1128/MMBR.00061-19. 32132243 PMC7062200

[43] Whelan S. 2013. Viral replication strategies, p 105–126. *In* Knipe DM, Howley PM, Cohen JI, Griffin DE, Lamb RA, Martin MA, Racaniello VR, Roizman B (ed), Fields virology, 6th ed, vol 1. Lippincott Williams & Wilkins, Philadelphia, PA.

[44] Wu Z, Han Y, Liu B, Li H, Zhu G, Latinne A, Dong J, Sun L, Su H, Liu L, Du J, Zhou S, Chen M, Kritiyakan A, Jittapalapong S, Chaisiri K, Buchy P, Duong V, Yang J, Jiang J, Xu X, Zhou H, Yang F, Irwin DM, Morand S, Daszak P, Wang J, Jin Q. 2021. Decoding the RNA viromes in rodent lungs provides new insight into the origin and evolutionary patterns of rodent-borne pathogens in Mainland Southeast Asia. Microbiome 9:18. doi:10.1186/s40168-020-00965-z. 33478588 PMC7818139

[45] Zhang C, Wang Z, Cai J, Yan X, Zhang F, Wu J, Xu L, Zhao Z, Hu T, Tu C, He B. 2020. Seroreactive profiling of filoviruses in Chinese bats reveals extensive infection of diverse viruses. J Virol 94:e02042-19. doi:10.1128/JVI.02042-19. 31941778 PMC7081923

[46] Sutton TDS, Clooney AG, Ryan FJ, Ross RP, Hill C. 2019. Choice of assembly software has a critical impact on virome characterisation. Microbiome 7:12. doi:10.1186/s40168-019-0626-5. 30691529 PMC6350398

[47] Sun Y, Qu Y, Yan X, Yan G, Chen J, Wang G, Zhao Z, Liu Y, Tu C, He B. 2022. Comprehensive evaluation of RNA and DNA viromic methods based on species richness and abundance analyses using marmot rectal samples. mSystems 7:e00430-22. doi:10.1128/msystems.00430-22. 35862817 PMC9426427

[48] Parras-Moltó M, Rodríguez-Galet A, Suárez-Rodríguez P, López-Bueno A. 2018. Evaluation of bias induced by viral enrichment and random amplification protocols in metagenomic surveys of saliva DNA viruses. Microbiome 6:119. doi:10.1186/s40168-018-0507-3. 29954453 PMC6022446

[49] Nayfach S, Camargo AP, Schulz F, Eloe-Fadrosh E, Roux S, Kyrpides NC. 2021. CheckV assesses the quality and completeness of metagenome-assembled viral genomes. Nat Biotechnol 39:578–585. doi:10.1038/s41587-020-00774-7. 33349699 PMC8116208

[50] Zolfo M, Pinto F, Asnicar F, Manghi P, Tett A, Bushman FD, Segata N. 2019. Detecting contamination in viromes using ViromeQC. Nat Biotechnol 37:1408–1412. doi:10.1038/s41587-019-0334-5. 31748692

[51] Sun T-W, Yang C-L, Kao T-T, Wang T-H, Lai M-W, Ku C. 2020. Host range and coding potential of eukaryotic giant viruses. Viruses 12:1337. doi:10.3390/v12111337. 33233432 PMC7700475

[52] Abergel C, Legendre M, Claverie J-M. 2015. The rapidly expanding universe of giant viruses: Mimivirus, Pandoravirus, Pithovirus and Mollivirus. FEMS Microbiol Rev 39:779–796. doi:10.1093/femsre/fuv037. 26391910

[53] Sahmi-Bounsiar D, Rolland C, Aherfi S, Boudjemaa H, Levasseur A, La Scola B, Colson P. 2021. Marseilleviruses: an update in 2021. Front Microbiol 12:648731. doi:10.3389/fmicb.2021.648731. 34149639 PMC8208085

[54] Hulo C, De Castro E, Masson P, Bougueleret L, Bairoch A, Xenarios I, Le Mercier P. 2011. ViralZone: a knowledge resource to understand virus diversity. Nucleic Acids Res 39:D576–D582. doi:10.1093/nar/gkq901. 20947564 PMC3013774

[55] Johnson WE. 2019. Origins and evolutionary consequences of ancient endogenous retroviruses. Nat Rev Microbiol 17:355–370. doi:10.1038/s41579-019-0189-2. 30962577

[56] Steinegger M, Söding J. 2017. MMseqs2 enables sensitive protein sequence searching for the analysis of massive data sets. Nat Biotechnol 35:1026–1028. doi:10.1038/nbt.3988. 29035372

