# Supplementary figures and images for: Elimination of Foreign Sequences in Eukaryotic Viral Reference Genomes Improves the Accuracy of Virome Analysis
Source: mSystems. 2022 Oct 26;7(6):e00907-22. doi: 10.1128/msystems.00907-22 (PMC9765019; doi:10.1128/msystems.00907-22)

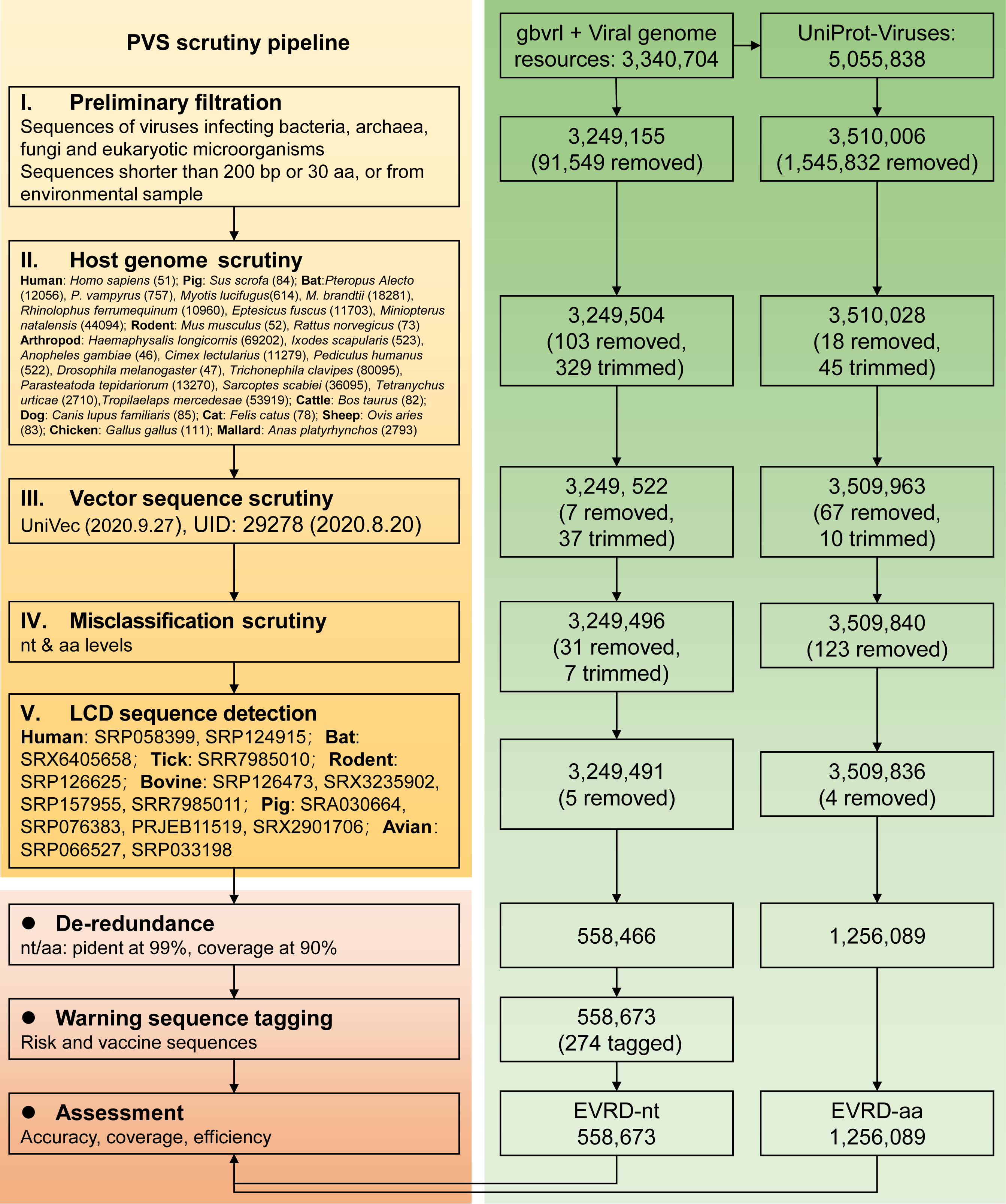

Supplement: FIG S1 [file msystems.00907-22-s0001.tif]

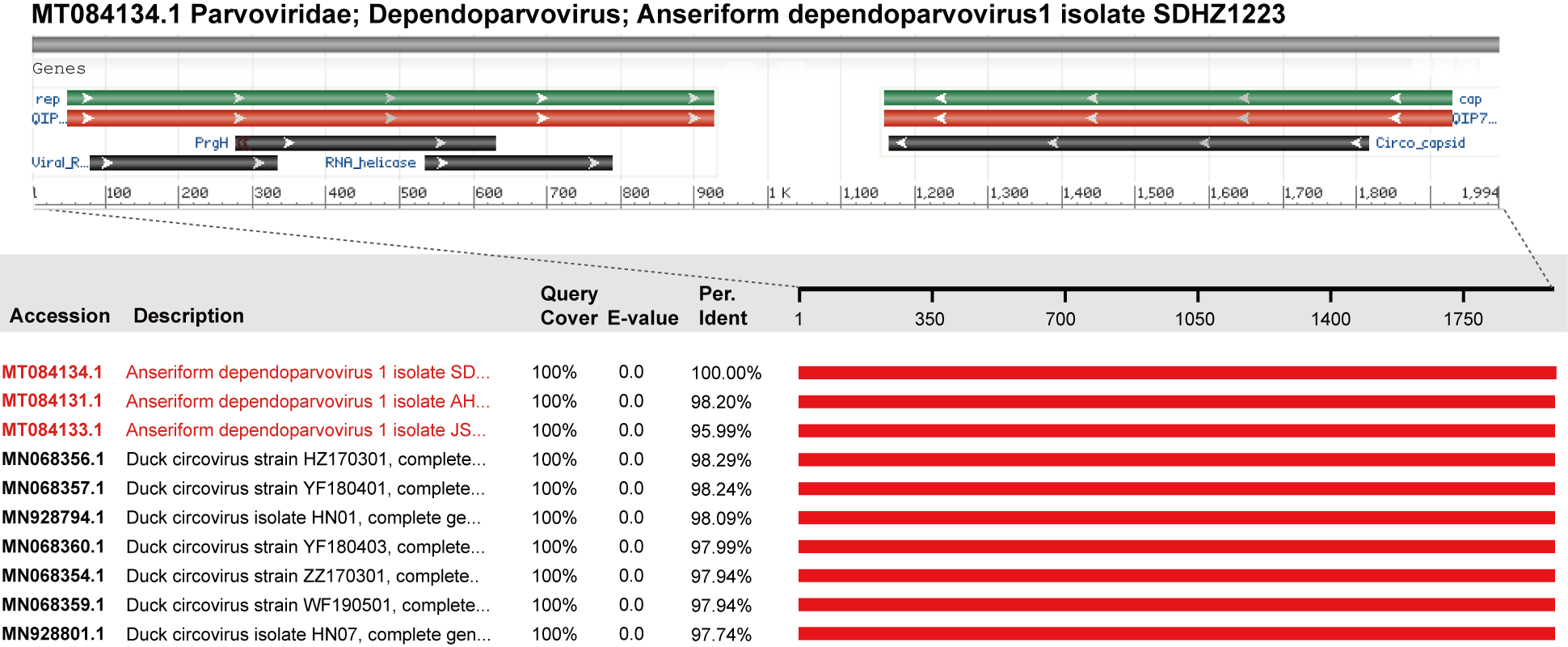

Supplement: FIG S2 [file msystems.00907-22-s0002.tif]

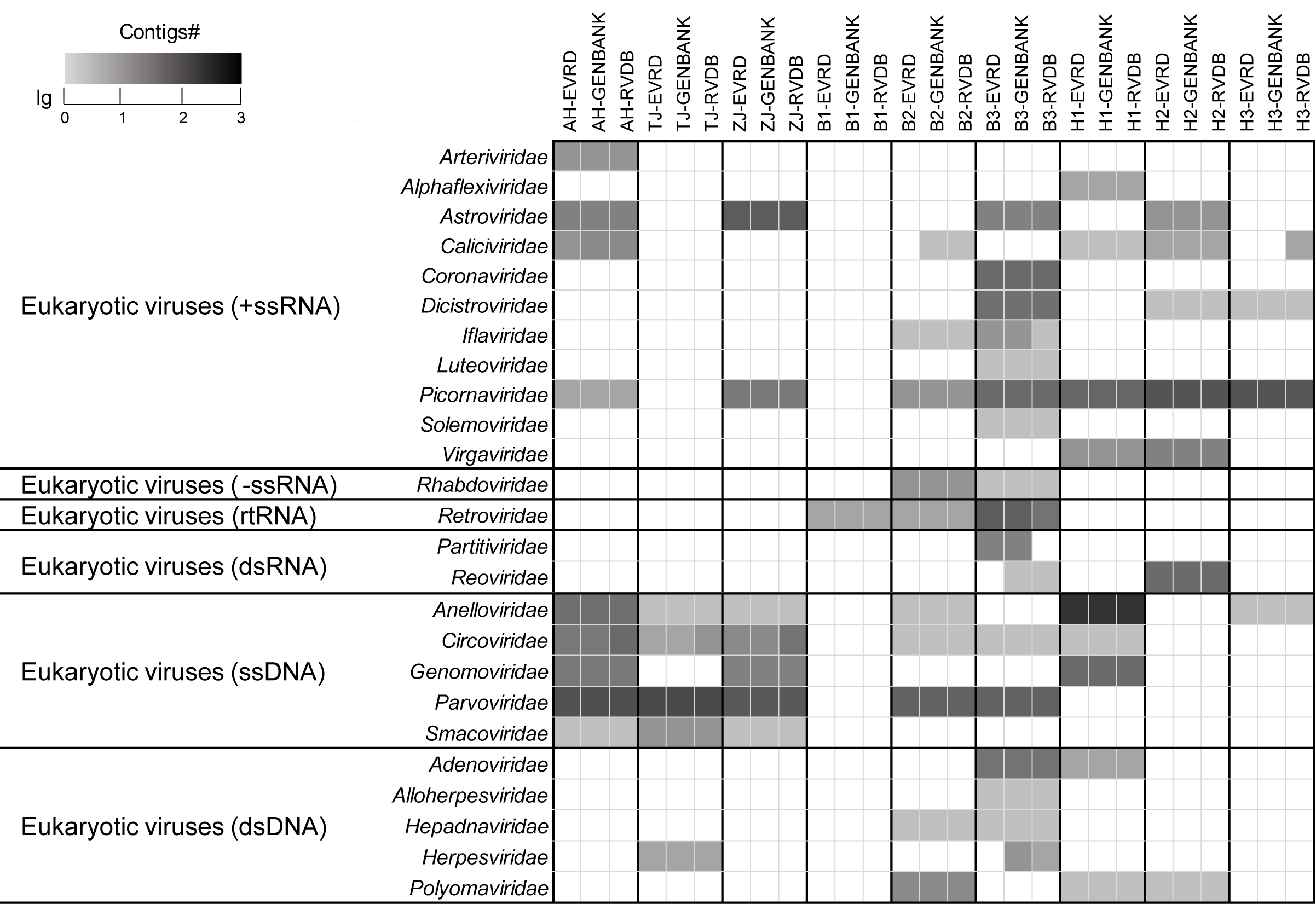

Supplement: FIG S4 [file msystems.00907-22-s0004.tif]

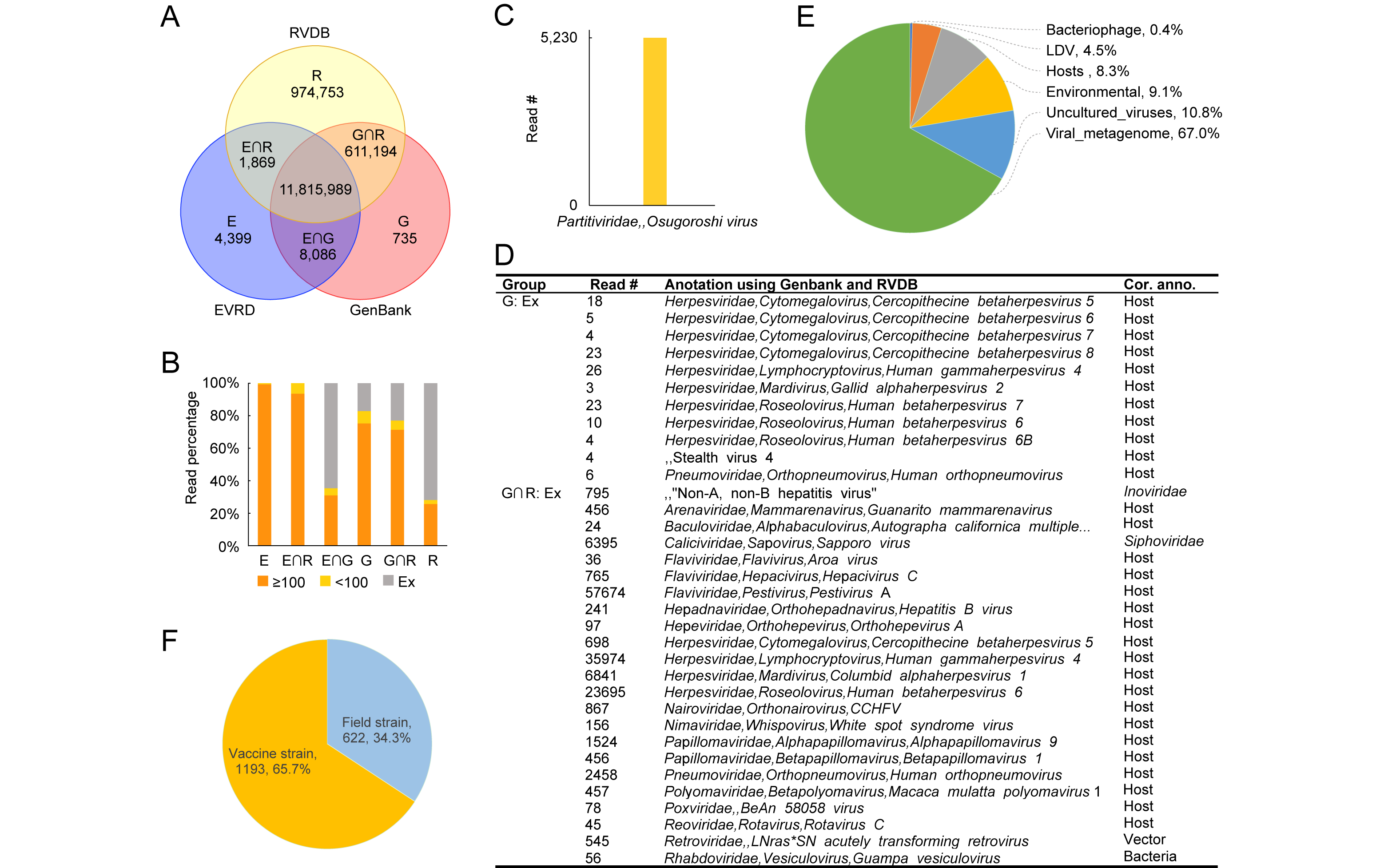

Supplement: FIG S5 [file msystems.00907-22-s0005.tif]

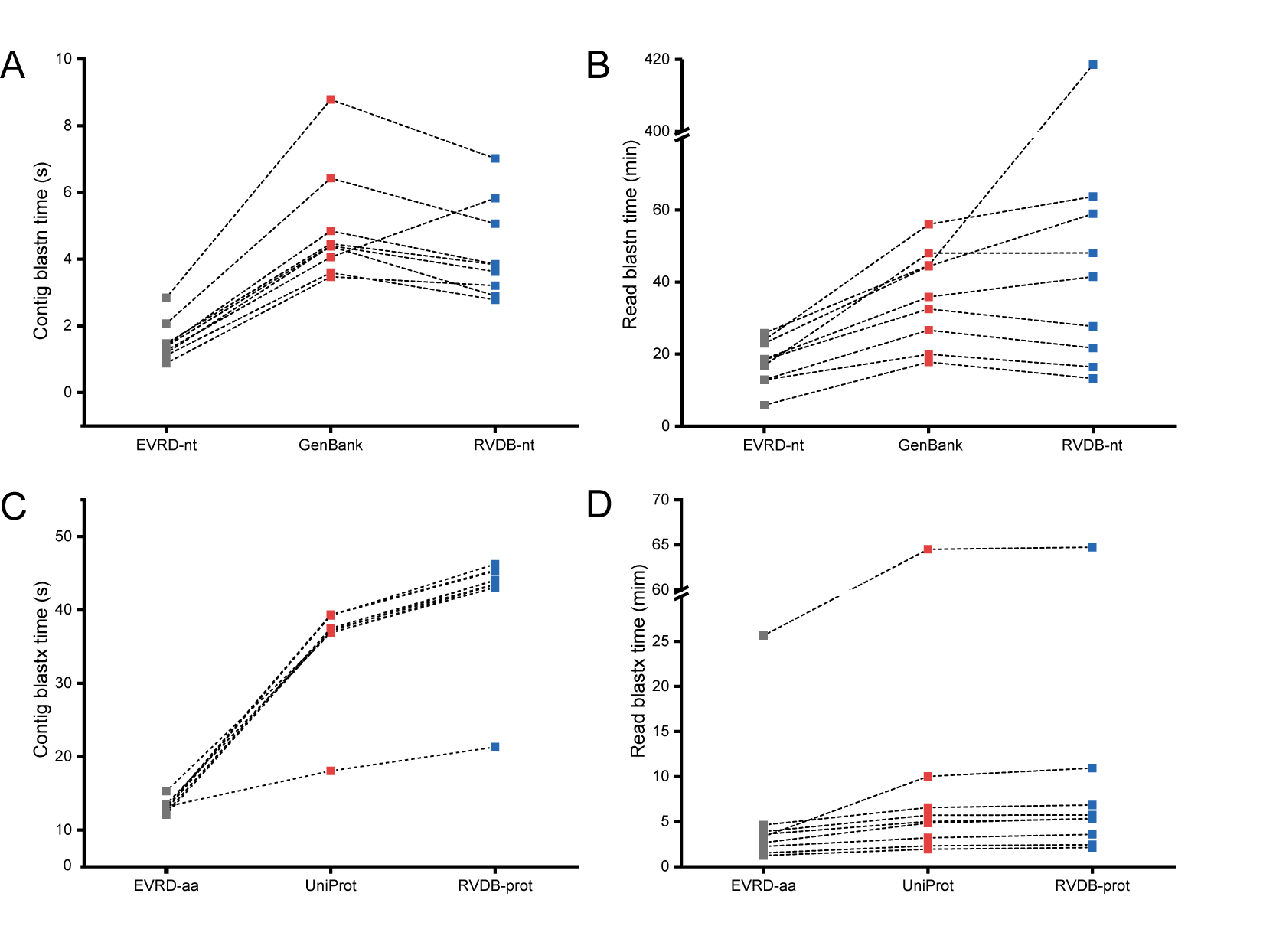

Supplement: FIG S6 [file msystems.00907-22-s0006.tif]
