# Supplementary material for: Elimination of Foreign Sequences in Eukaryotic Viral Reference Genomes Improves the Accuracy of Virome Analysis
Source: mSystems. 2022 Oct 26;7(6):e00907-22. doi: 10.1128/msystems.00907-22 (PMC9765019; doi:10.1128/msystems.00907-22)
Supplement: TEXT S1 [file msystems.00907-22-s0010.docx]

## Supplementary Text

**Scrutiny pipeline for viral protein sequences**

The protein sequences retrieved from UniProt virus division were subjected to scrutiny of nt database as described in main text with minor modification. In the step of preliminary filtration, we removed those non-eukaryotic viral sequences and those ≤ 30 aa. The remaining sequences were used to blastp search against the genomic protein sequences of the hosts to detect any potential host contaminants (length ≥ 100 and identity ≥ 90%), these host contaminants if detected were further subjected to blastp search against nr database to finally identify whether they are host protein sequences with the same criterion used in nt identification. The scrutiny was iteratively performed until no host contaminants were found. In the vector sequence scrutiny, we generated a non-viral protein core (NVPC) that consists of nonviral expression elements (n=13,287) born in vectors. To achieve that, those protein sequences ≥ 100 aa encoded by vectors were de-replicated using cd-hit v4.8.1 with 99% similarity at 90% coverage for the shorter sequences (1). The resulting representatives (n=17,236) were blastp searched against the nr database using Diamond with maximum number of 100 target sequences to report alignments (2). The representatives classified as viruses using a majority-rules approach were discarded, while the rest (n=15,220) were further queried against the UniProt viruses branch. These unaligned sequences (n=12,603) were technically nonviral and classified into NVPC, while these aligned (n=2,617) were manually inspected by online blastx search against nr database with these (n=684) annotated to nonviral products being classified into NVPC. And then, a blastp search of PDS against NVPC was conducted to find any vector contaminants. The queries with identity ≥90% over alignment ≥100 with NVPC were further validated and treated as described in host protein scrutiny. The annotation cross scrutiny of viral protein sequences was nearly the same as that in nt scrutiny but only that the all-against-all blastp hits were considered significant if their e-values were ≤ 1e-50 and length ≥ 100. In cross check of the viral metagenomes, contigs ≥ 1000 bp were subjected to blastx search against viral protein sequences. The viral protein sequences were considered suspicious if they matched to contigs of viral metagenomes from ≥ two host species, and subjected to further validation by blastp search against nr database as described in cross check of the viral metagenomes.

**Overview of heterogenous sequences**

The submission of these PVSs could be traced back to 1993 with 66.2% from 2015-2019 (Fig. 1 and Data set S1). HTS-based viral metagenomics has dramatically expanded the space of our known viral sequences (3), but with an unwanted side-effect, i.e., the chimeric viral assembly containing insertion of other viral sequences, even sequences of other organisms (4). Though a lot of PVSs did not provide the information of sequencing technology in GenBank, we did find a substantial number of host PVSs (n > 51) submitted since 2015 are probably due to the *de novo* assembly of Illumina reads.

**EVRD improves the accuracy and efficiency of viromic analysis**

The performance of EVRD was evaluated in viromic analysis by comparison of its ability to avoid false positives, possibility to miss true viral contigs, and time to complete the analysis with Genbank (for nt) and UniProt (for aa) viral branches, and RVDB (v21.0) using nine viral metagenomic data of pigs, bats and humans (Table S1). The results at the read level revealed that 13,417,025 reads in the nine datasets were annotated to be viruses by at least one of the databases, covering 47 families with 15 exclusively invisible to EVRD-nt in some datasets (Fig. 5). Majority (88.1%) of these virus-like reads (VLRs) were co-annotated by them, suggesting a high consistency using the three databases (Figs. 5 and S5A). Among those inconsistently annotated VLRs, 60.9% were exclusively annotated by RVDB-nt (subset R in Fig. S5A), followed by 38.2% being co-annotated by RVDB-nt and GenBank (G∩R in Fig. S5A).

The criterion used to determine whether a sequence is viral substantially impacts the annotation of these inconsistent reads. Some of these HTS datasets were generated with an insert size of 125 bp (Table S1), so the requirement of alignment length ≥ 120 is a little stringent to them and has excluded many true positives. If we loosened the length cutoff to 100, such consistency was variably improved (Fig. S5B). Almost all of VLRs in subsets E and E∩R were annotated by the other database(s) using a loose length cutoff (Fig. S5B). But there were still lots of reads unable to be annotated by certain database(s) even using a loose length cutoff (illustrated using Ex in Fig. S5B). After improvement, 5,230 VLRs in E∩G remained unable to be annotated by RVDB-nt. All of these reads were related to Osugoroshi viruses within the family *Partitiviridae* that were recently released to the public by GenBank and have yet been synchronized in RVRD-nt v21.0 (Fig. S5C). The Ex VLRs in subsets G and G∩R, and their *de novo* assemblies, were all annotated to PVSs (Fig. S5D), i.e., they were false positives. The overwhelming majority (95.5%) of Ex VLRs in subsets R were related to sequences that are unrelated to eukaryotic viral pathogens and exclusively recruited by RVDB-nt, i.e., viral metagenomes, uncultured viruses, environmental samples, host-derived endogenous viral elements and bacteriophage (Fig. S5E). The remaining 4.5% were related to microorganism-infecting LDVs, such as pandora viruses and pithoviruses (Fig. S5E).

*de novo* assemblies (≥ 1000 bp) were also annotated using these databases. Compared to the results revealed using reads, 22 viral families were lost (Fig. S6) including *Filoviridae* that has proved to be present in samples (5). The annotation using EVRD-nt excluded the false positives of *Caliciviridae*, *Reoviridae* and *Herpesviridae* in certain datasets, indicating an improvement of accuracy at the contig level. Though the annotation using aa references of the three databases all showed higher specificity at the read and contig levels, EVRD-aa improved more significantly with exclusion of the false positives from *Reoviridae*, *Parvoviridae* and *Mitoviridae*, etc. These results indicated that the de-heterogeneity of our EVRD does not sacrifice the detection spectrum of eukaryotic viruses, but rather significantly improves the specificity and accuracy of viromic annotation via reduction of erroneous annotation.

We did not find any viromic annotations tagged with ‘LCD’ or ‘Vector’, indicating no contamination of laboratory component- and vector-derived sequences in these datasets. But of special note is that, besides 622 reads in dataset AH annotated to porcine reproductive and respiratory syndrome virus (PRRSV) field strains, there were another 1,193 reads annotated to PRRSV vaccine strain in the dataset (Fig. S5F), indicating co-circulation of field viruses and vaccine strains in the farm, which should be especially concerning, since new viruses could be generated through recombination between field viruses and vaccine strains, resulting in vaccine failure (6). Viromic annotation is quite time- and computing resource-consuming. A small-scale reference database can shorten the analytic time and minimize the computing resource. With an entry-level platform, analyses of reads or contigs at nt or aa levels using EVRD were 1.8-3.3 and 1.9-3.2 times faster than using GenBank/UniProt and RVDB, respectively (Fig. S4), indicating that EVRD is more efficient.

**References**

1. Fu L, Niu B, Zhu Z, Wu S, Li W. 2012. CD-HIT: accelerated for clustering the next-generation sequencing data. Bioinformatics 28:3150-3152.

2. Buchfink B, Xie C, Huson DH. 2014. Fast and sensitive protein alignment using DIAMOND. Nat Methods 12:59-60.

3. Simmonds P, Adams MJ, Benkő M, Breitbart M, Brister JR, Carstens EB, Davison AJ, Delwart E, Gorbalenya AE, Harrach B, Hull R, King AMQ, Koonin EV, Krupovic M, Kuhn JH, Lefkowitz EJ, Nibert ML, Orton R, Roossinck MJ, Sabanadzovic S, Sullivan MB, Suttle CA, Tesh RB, van der Vlugt RA, Varsani A, Zerbini FM. 2017. Virus taxonomy in the age of metagenomics. Nat Rev Microbiol 15:161-168.

4. Roux S, Adriaenssens EM, Dutilh BE, Koonin EV, Kropinski AM, Krupovic M, Kuhn JH, Lavigne R, Brister JR, Varsani A, Amid C, Aziz RK, Bordenstein SR, Bork P, Breitbart M, Cochrane GR, Daly RA, Desnues C, Duhaime MB, Emerson JB, Enault F, Fuhrman JA, Hingamp P, Hugenholtz P, Hurwitz BL, Ivanova NN, Labonté JM, Lee K-B, Malmstrom RR, Martinez-Garcia M, Mizrachi IK, Ogata H, Páez-Espino D, Petit M-A, Putonti C, Rattei T, Reyes A, Rodriguez-Valera F, Rosario K, Schriml L, Schulz F, Steward GF, Sullivan MB, Sunagawa S, Suttle CA, Temperton B, Tringe SG, Thurber RV, Webster NS, Whiteson KL, et al. 2019. Minimum Information about an Uncultivated Virus Genome (MIUViG). Nat Biotechnol 37:29-37.

5. Zhang C, Wang Z, Cai J, Yan X, Zhang F, Wu J, Xu L, Zhao Z, Hu T, Tu C, He B. 2020. Seroreactive profiling of filoviruses in Chinese bats reveals extensive infection of diverse viruses. J Virol 94:e02042-19.

6. He B, Gong W, Yan X, Zhao Z, Yang Le, Tan Z, Xu L, Zhu A, Zhang J, Rao J, Yu X, Jiang J, Lu Z, Zhang Y, Wu J, Li Y, Shi Y, Jiang Q, Chen X, Tu C. 2021. Viral metagenome-based precision surveillance of pig population at large scale reveals viromic signatures of sample types and influence of farming management on pig virome. mSystems 6:e00420-21.
